# Phenotypic Characterization of Prostate Cancer LNCaP Cells Cultured within a Bioengineered Microenvironment

**DOI:** 10.1371/journal.pone.0040217

**Published:** 2012-09-05

**Authors:** Shirly Sieh, Anna V. Taubenberger, Simone C. Rizzi, Martin Sadowski, Melanie L. Lehman, Anja Rockstroh, Jiyuan An, Judith A. Clements, Colleen C. Nelson, Dietmar W. Hutmacher

**Affiliations:** 1 Regenerative Medicine and Cancer Program, Institute of Health and Biomedical Innovation, Queensland University of Technology, Brisbane, Queensland, Australia; 2 Australian Prostate Cancer Research Centre-Queensland, Queensland University of Technology and Princess Alexandra Hospital, Woolloongabba, Queensland, Australia; 3 Department of Urologic Sciences, Vancouver Prostate Centre, University of British Columbia, Vancouver, British Columbia, Canada; University of North Carolina at Chapel Hill, United States of America

## Abstract

Biophysical and biochemical properties of the microenvironment regulate cellular responses such as growth, differentiation, morphogenesis and migration in normal and cancer cells. Since two-dimensional (2D) cultures lack the essential characteristics of the native cellular microenvironment, three-dimensional (3D) cultures have been developed to better mimic the natural extracellular matrix. To date, 3D culture systems have relied mostly on collagen and Matrigel™ hydrogels, allowing only limited control over matrix stiffness, proteolytic degradability, and ligand density. In contrast, bioengineered hydrogels allow us to independently tune and systematically investigate the influence of these parameters on cell growth and differentiation. In this study, polyethylene glycol (PEG) hydrogels, functionalized with the Arginine-glycine-aspartic acid (RGD) motifs, common cell-binding motifs in extracellular matrix proteins, and matrix metalloproteinase (MMP) cleavage sites, were characterized regarding their stiffness, diffusive properties, and ability to support growth of androgen-dependent LNCaP prostate cancer cells. We found that the mechanical properties modulated the growth kinetics of LNCaP cells in the PEG hydrogel. At culture periods of 28 days, LNCaP cells underwent morphogenic changes, forming tumor-like structures in 3D culture, with hypoxic and apoptotic cores. We further compared protein and gene expression levels between 3D and 2D cultures upon stimulation with the synthetic androgen R1881. Interestingly, the kinetics of R1881 stimulated androgen receptor (AR) nuclear translocation differed between 2D and 3D cultures when observed by immunofluorescent staining. Furthermore, microarray studies revealed that changes in expression levels of androgen responsive genes upon R1881 treatment differed greatly between 2D and 3D cultures. Taken together, culturing LNCaP cells in the tunable PEG hydrogels reveals differences in the cellular responses to androgen stimulation between the 2D and 3D environments. Therefore, we suggest that the presented 3D culture system represents a powerful tool for high throughput prostate cancer drug testing that recapitulates tumor microenvironment.

## Introduction

Prostate cancer (CaP) is one of the most prevalent malignant diseases among men in western countries. The 5-year survival rate for men diagnosed with localized CaP approaches 100%; whereas, the prognosis worsens rapidly upon CaP progression to advanced and metastatic disease [1]–[2]. Despite advancement in detection methods and treatments, CaP remains a major cause of cancer death in men. Therefore, it is important to gain a greater understanding of the progression from localized to advanced CaP using relevant physiological systems. Like many other cancer cells, CaP cells have been extensively studied in two dimensional (2D) cultures, through which a significant basic understanding of cancer biology has been gained. However, in native tissues, cells are embedded in extracellular matrix (ECM) that provides not only architectural support, but also chemical and mechanical cues to cells [3]–[4]. Recently, the importance of the mechanical properties of the tumor microenvironment has been increasingly acknowledged. In general, the cancerous tissue and its stroma are stiffer than non malignant tissues due to abnormal deposition and remodeling of the ECM in the stroma [5]–[7]. *In vitro* studies and few *in vivo* studies have demonstrated that stiffness of the surrounding matrix can increase cancer cell growth, modulate cell signaling and facilitate cell invasion [8]–[11]. Considering the vital role of matrix rigidity, artificial geometric constraints and the high stiffness imposed on cells on 2D tissue culture plastic could affect tumor growth, adhesion, cell polarity, morphology, migration and proteolysis mechanisms [12]–[15].

In recent years, numerous studies have demonstrated that studying tumors in 3D better reproduces *in vivo* growth characteristics and resistance against chemotherapeutic agents than a 2D approach [16]–[18]. The most commonly used 3D matrix models are animal derived reconstituted basement membrane extract, Matrigel™ [17], [19]–[20], and rat tail collagen type I matrices [21]–[24]. Although these naturally derived matrices have ECM-like biological properties, their inherent characteristics limit the flexibility of adjusting matrix stiffness without simultaneously affecting other matrix properties such as proteolytic degradability and ligand density. Furthermore, Matrigel™ shows batch-to-batch variations, which decreases the reproducibility of experiments and comparability of data sets between different laboratories. To simplify the otherwise complex cellular interactions of the multiple components which occur in naturally derived matrices, there is a growing interest in the use of matrices engineered with specific biological and biochemical features of natural ECM [25]–[28]. These biomimetic matrices allow a more systematic study of the impact of certain components or properties of the tumor microenvironment on cancer cells. Therefore, emerging approaches in biomaterial science have focused on the development of synthetic matrices such as hyaluronon-derived or alginate matrices for culturing cancer cells [25]–[28]. Another example is PEG-based hydrogels that are inert themselves in terms of triggering cell signaling pathways, but can be equipped with biochemical (e.g. proteolytic degradation sites) and biological functionalities (e.g. RGD motifs in a controlled fashion) [29]. Advantageously, the stiffness of such hydrogels can be precisely tuned, independent of their proteolytic sensitivity and cell ligand density.

Previously, we and others have used MMP-sensitive PEG-based hydrogels in which RGD motifs are incorporated at a defined density [13], [30]–[32]. The RGD motifs provide binding sites for cells via integrins, and the MMP cleavage sequences permit cells to degrade the matrix, which creates space for cell proliferation and migration. To this end, a thorough phenotypic characterization of CaP cells cultured within this biomimetic ECM has not been reported. In this study, we aimed to establish and validate a 3D culture system for LNCaP cells that allows modeling of the early stage avascular tumor formation. To do this, we investigated the effect of the hydrogel stiffness on cell growth. Thereafter, we examined morphology, gene expression and protein synthesis of LNCaP cells grown in 3D hydrogels compared to conventional 2D cultures. We also used this 3D culture system to study the effects of the synthetic androgen, R1881, on AR signaling in comparison to 2D cultures [33]–[34].

Our findings provide insights into the role of the microenvironment in modulating the cellular and molecular behavior of cancer cells. Furthermore, we reveal differences in cellular responses to androgens between 2D and 3D cultures. This model is a stepping stone for the development of 3D culture systems replicating the *in vivo* tumor microenvironment, allowing for the creation of powerful tools to better understand CaP biology, in particular in response to androgens.

## Results

### Mechanical and diffusive properties of biomimetic hydrogels are dependent on the PEG content

**Figure 1:**
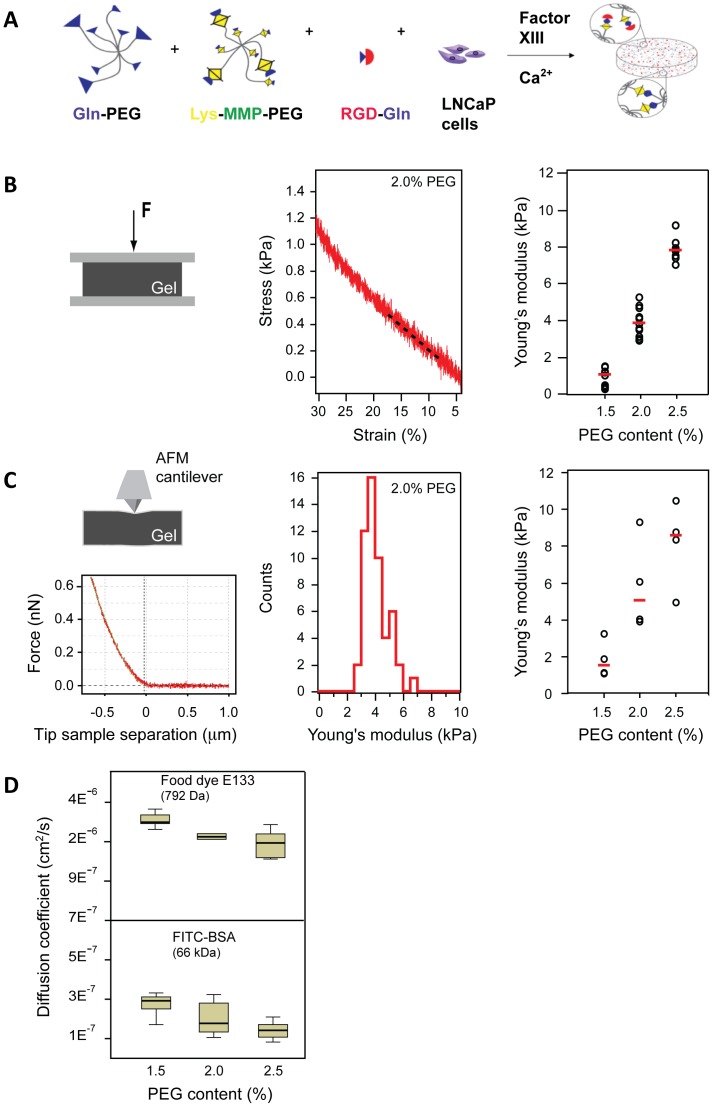
Characterization of PEG-based biomimetic hydrogels. (A) A schematic showing the preparation of biomimetic hydrogels. LNCaP cells were mixed with PEG precursors containing MMP cleavage sites and RGD peptides prior to FXIII-catalyzed polymerization. (B) Global stiffness of cell-free hydrogels with different PEG content (w/v) was measured using a microtester. A representative stress-strain curve recorded for a 2% PEG gel. The (elastic) Young's modulus, E, was calculated from the slope of the line fitted to the stress-strain curve at 9–12% strain (middle). A Scatter plot showing the Young's moduli measured for different PEG hydrogels (right). Medians are indicated by the red bars. (C) Local stiffness of cell-free hydrogels with different PEG content (w/v) was measured using atomic force microscopy (AFM). A pyramidal shaped AFM cantilever was used to conduct indentation measurements. A representative force versus tip-sample-separation curve is shown below the AFM schematic. The red line represents the fit used to approximate the approach curve with a Hertzian indentation model. Young's moduli measured for a 2% PEG hydrogel is normally distributed in the range of 4 kPa (middle). Scatter plot shows the median Young's moduli measured for different hydrogels (right). Red bars represent the medians. Both microtester and AFM indentation measurements yield similar Young's moduli and show a linear increase in stiffness with PEG content. (D) Box plots show the diffusion coefficients, D measured for the food dye E133 (794 Da) and FITC-BSA (66 kDa) in cell-free hydrogels at different PEG content. Values of D for E133 are one order of magnitude greater than for FITC-BSA. At least three samples were measured from three independent experiments.

To mimic the early stage of CaP tumor formation in 3D, we set out to culture LNCaP cells in biomimetic PEG hydrogels. The PEG precursors conjugated with MMP cleavage sites and RGD motifs were incorporated into the hydrogel network to confer essential biomimetic features of the natural ECM (Figure 1A). Since mechanical properties of the hydrogel are known to influence growth, morphology, and invasive phenotype of cancer cells [5], [10], [35], we first aimed to optimize the hydrogel's stiffness for cell culture. Cell-free PEG hydrogels of varying stiffness were prepared by adjusting the PEG content to 1.5, 2, 2.5% (w/v). The stiffness of the hydrogel was determined by unconfined compression tests using a microtester (Figure 1B, left) and atomic force microscopy (AFM) indentation measurements (Figure 1C, left) which were converted to a stress-strain curve (Figure 1B, middle).

While the microtestor probes the global elastic properties of the PEG hydrogels, the AFM indentation measurements yield information about their local stiffness distribution (Figure 1C). Both methods revealed a linear increase in elastic moduli with augmentation of PEG content and indicated similar elastic moduli, ranging between approximately 0.8 kiloPascal (kPa) to 10 kPa for the 1.5% and 2.5% PEG hydrogels, respectively (Figure 1B and 1C, right). AFM measurements revealed a homogenous distribution of local Young's modulus, E over the probed hydrogels (Figure 1C, middle). When the 2.0% hydrogels were tested using the microtester before and after more than 4 weeks of culture, we found no significant changes in the overall matrix stiffness (2.5–4.2 kPa), which was consistent with the variation in the tested 2% hydrogels (Figure S1). As previously reported in 3D cell cultures within similar matrices [5], [10], [35], this indicates that these biomimetic PEG-based hydrogel are only locally degraded by the embedded cells, while the overall stiffness is not affected over the analyzed growth period.

Changes in PEG content are expected to alter the pore size of the PEG hydrogels [36] which could affect the transfer of small molecules, such as the synthetic androgen R1881, and also growth factors present in the medium in our experiments. To estimate the diffusion of R1881, we measured the diffusion coefficient D, of the food dye E133 (792 Da), that has a higher molecular weight than R1881 (284 Da). We found that the median values of diffusion decreased with increasing PEG content (Figure 1D) from 2.99±0.06×10^−6^ cm^2^/s for 1.5% PEG hydrogels to 1.9±0.13×10^−6^ cm^2^/s for 2.5% PEG hydrogels. To test if essential growth factors such as Fibroblast growth factor, Vascular endothelial growth factor and Platelet-derived growth factor (20–50 kiloDalton, kDa) can also penetrate the hydrogel, we investigated the diffusion of Fluorescein isothiocyanate-conjugated bovine serum albumin (FITC-BSA) (66 kDa), which has a higher molecular weight than these growth factors. The diffusion coefficient of FITC-BSA was lower compared to E133 and decreased with increasing PEG content (Figure 1D). D values for 1.5–2.5% PEG hydrogels (1.5–3.0×10^−7^ cm^2^/s) are within the same order of magnitude (6×10^−7^ cm^2^/s) reported by Ramanujan *et al.* (2002) and Erikson *et al.* (2008) measuring diffusion of BSA in the softer collagen gels [37]–[38]. When PEG hydrogels were incubated with FITC-BSA for 24 hours, we observed widespread penetration of the FITC-BSA (Figure S2). From these findings we may conclude that, although transfer of R1881 and growth factors may be slowed down in gels with higher PEG content, the pore size of all gels is sufficiently large to provide embedded cells with soluble factors from the culture medium.

### Proliferation of LNCaP cells within biomimetic hydrogels is influenced by the PEG content and stiffness of hydrogels

**Figure 2:**
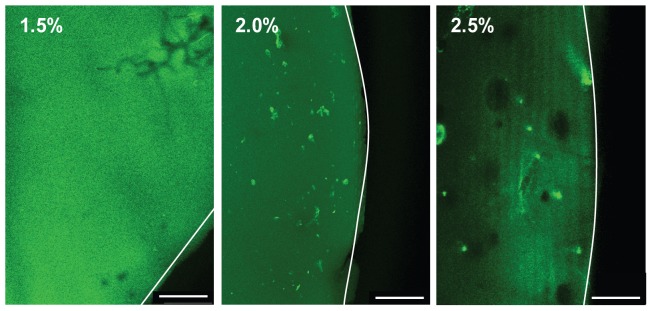
Characterization of LNCaP cell growth cultured within biomimetic hydrogels in normal growth media, RPMI and 10% FBS. (A) Growth curves of LNCaP cells over 7 days for 2D cultures (left) and 28 days for 3D cultures (right) in normal growth media measured by total DNA content (mean ± SE). In 2D cultures cells proliferate much faster reaching confluency within 7 days (top panel, left). The growth profile of cells is dependent on the mechanical properties of the bioengineered PEG matrices, where growth rate is highest in 1.5% followed by cells grown in 2% hydrogels (top panel, right). No cell proliferation is detected in the stiffer hydrogels (2.5%). Live-dead examination was performed using fluorescein diacetate-propidium iodide staining on day 24 of LNCaP cells grown in different PEG content (lower panel). The live-dead staining reveals that most cells are viable (green) in both 1.5 and 2.0% hydrogels but not viable (red) in 2.5% hydrogels. Images were taken with CLSM (10×, 0.4 NA). Bright field images also confirm that cells in 2.5% hydrogels did not form colonies as observed in 1.5% and 2.0% hydrogels at day 28. (B) (top panel) A representative CLSM 3D projections of LNCaP colonies labeled with Phalloidin (actin, red)/DAPI (nuclei, blue) from day 7 to day 28 (20×, 1.0 NA). Colonies are more rounded at day 7 and day 14 but become irregular aggregates from day 21 onwards. Box plots of colony size and shape factor analyzed from six CLSM images (bottom panel) show significant increase in spheroid size and decrease in shape factor (p<0.001) at day 28 relative to day 7 cultures are detected. (C) A representative histology section of day 28 cultures reveal the formation of invasive ‘fingers’ (arrow) penetrating the surrounding hydrogel (left). The H&E stain (middle) shows a hollow core. Immunohistochemistry stain of Caspase 8 (right) confirms apoptotic cells within the core (brown). (D) Scatter plots of colony size, cell number and core size if any analyzed from 10 µm thick histology sections. This shows that each colony size increases linearly with the cell number. Core size also increases linearly with the colony size. At least three independent experiments were conducted for each analysis. Scale bars: (A, left, B) 250 µm, (A, right) 100 µm and (C) 50 µm.

Next, to evaluate the effect of matrix rigidity on cell proliferation, we cultured LNCaP cells in 1.5, 2.0 and 2.5% hydrogels over 28 days (Figure 2A, top panel, right). Monolayer cultures (doubling time, Td = 27 hr) grew exponentially up to day 5 and began to plateau thereafter (Figure 2A, top panel, left). On the contrary, cells proliferated slower in 1.5% (Td = 54 hr) and 2% (Td = 58.5 hr) PEG hydrogels. Exponential growth occurred between day 7 and 14 in 3D cultures (Figure 2A, top panel, right). LNCaP cells continued to proliferate at least up to 28 days in the 2.0% hydrogels, while maximum cell numbers were reached after 14 days in the 1.5% hydrogels. The higher growth rates in the softer 1.5% hydrogel compared to the 2.0% hydrogel may be due to the lower stress exerted on the cells by the surrounding softer hydrogel [39]–[40]. The sigmoidal growth curve we observe is comparable to the observed growth of LNCaP xenografts with evidence of slow growing at the early stage, exponential growth in the intermediate stage and slowing down of growth at the later stage [41]–[46]. On the other hand, no growth was detected in 2.5% hydrogels as confirmed by the live-dead staining (Figure 2A, bottom panel) revealing the presence of predominantly non viable cells in the 2.5% hydrogel at day 24. Bright field images showed that cells were able to form colonies in the 1.5 and 2.0%, but not in 2.5% hydrogels.

Based on this proliferation study, we have selected 2.0% PEG hydrogels for conducting subsequent experiments. To date, the local stiffness within the prostate glandular tissue is unknown. However, the stiffness of the 2% gels of 4 kPa are within the reported range of soft tissues (1–10 kPa) and breast glandular tissue (4 kPa) [5], [47].

### LNCaP spheroids in biomimetic hydrogels vary morphologically and phenotypically from LNCaP cells in 2D cultures

Next, we examined the morphology of LNCaP cells grown for up to 28 days in 2% biomimetic PEG hydrogels. Confocal images of Phalloidin and 4′,6-diamidino-2-phenylindole (DAPI) stained hydrogels (Figure 2B, top panel and Figure S3, left) and time lapse videomicroscopy (Video S1) showed that single cells proliferated into colonies up to 200 microns in diameter in the hydrogel. These colonies resembled the morphology of LNCaP colonies cultured in Matrigel™ (Figure S3, right) as we and others have previously described [48]–[49]. The colony size increased from day 7 to day 21 and remained constant thereafter (Figure 2B, bottom panel). The cell aggregates were spherical in shape up to day 21 subsequently becoming irregular, as confirmed by a significant decrease in shape factor at day 28 (Figure 2B, bottom panel). The Phalloidin-DAPI and Haematoxylin and Eosin (H&E) stainings of frozen sections revealed that the day 28 colonies formed ‘finger-like’ structures and often presented a hollow central core (Figure 2C). The presence of apoptotic cells in this region, as detected via Caspase 8 staining, was also observed. This increased number of apoptotic cells within the core which has been also described in other *in vitro* 3D tumor cultures [17], [19]. Further histological examination of the 3D cultures supports that the increase in size of each colony was proportionate to the cell number and size of cores formed (Figure 2D). Apoptotic core formations were more frequently observed in day 28 cultures compared to the other time points (data not shown).

**Figure 3 pone-0040217-g003:**
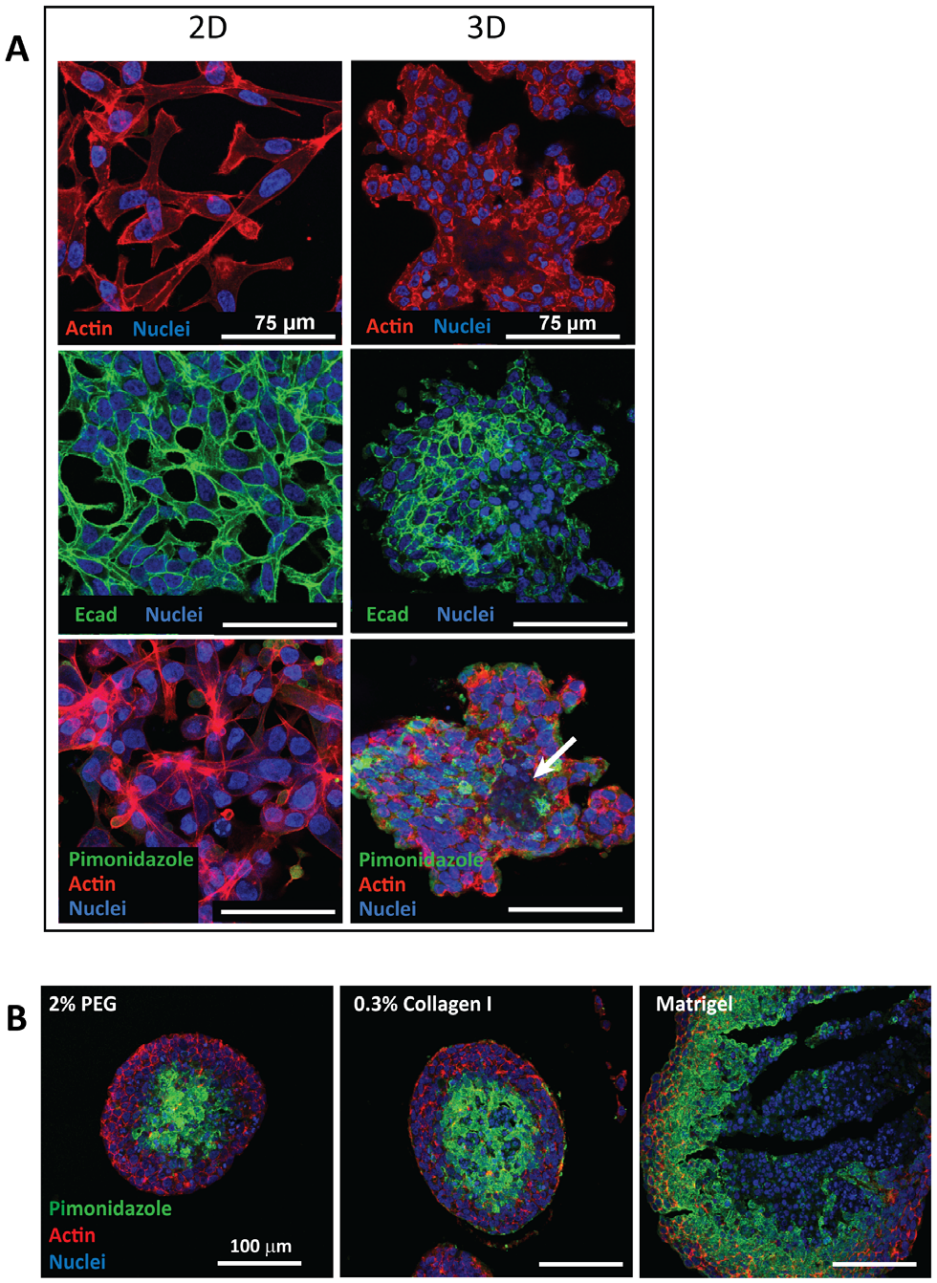
Immunofluorescent staining of LNCaP cells cultured in normal growth media from 2D and 3D cultures. (A) A comparison of 2D cultures (day 6) and histology sections of 3D cultures (day 28) reveals differences in cell morphology and phenotype. Cytoskeletal organization (actin, red) of cells in 2D cultures reveals a spread out and elongated cell morphology (top panel). The cell and nucleus (blue) size of 2D cultures is larger than the 3D cultures. In 3D cultures, cells adopt a cobblestone morphology and compact cell arrangement. They also form extensive cell-cell contact within the colony. Epithelial marker, E-cadherin (Ecad, green) is localized in the cell membrane and in cell-cell contacts for both cultures (middle panel). Hypoxia staining with Pimonidazole (green) indicates that there are less hypoxic cells in 2D cultures relative to 3D cultures (bottom panel). Hypoxia is detected within the LNCaP colony in the presence of an apoptotic core (arrow). (B) Pimonidazole stainings performed on LNCaP cells cultured in the PEG hydrogels, Collagen I and Matrigel™ for 24 days show presence of hypoxia in all the matrices. CLSM images were taken at 40× magnification and 1.25 NA. Scale bars: (A) 75 µm and (B) 100 µm.

Since most of the previous characterization of the LNCaP cell phenotype is based on 2D cultures, we set out to examine the 3D cultured LNCaP cell morphology. As shown in Figure 3A, LNCaP cells on 2D were spread out with spindle-like morphology. In the 3D cultures however, cells were arranged in compact cell aggregates, whereupon single cells adopted a more rounded morphology compared to monolayer cells. Within the LNCaP colony, the epithelial marker E-cadherin (Ecad) co-localised with cell-cell contacts, whereas in 2D cultures Ecad was more uniformly distributed on the cell surface. We next performed staining for hypoxia marker with Pimonidazole in both cultures to evaluate the oxygen availability to cells, detecting a higher hypoxia level in 3D than in 2D cultures. This observation is consistent with tumor xenografts and clinical tumors where hypoxia and central apoptosis/necrosis is a common feature in poorly vascularized solid tumors and previous 3D spheroid cultures independent of cell types [42], [50]–[54]. Our report also showed that in the earlier 3D cultures (day 14), where spheroid size was smaller (Figure S4), less Pimonidazole staining was evident compared to day 28 cultures (Figure 3). This suggests that multicellular aggregates, depending on the colony size can influence oxygen transfer within the colony. Similarly, when LNCaP cells were cultured in softer gels (e.g Collagen I and Matrigel™), hypoxia was detected with Pimonidazole staining at day 24 of culture growth (Figure 3B). Spheroids in suspension cultures also encounter oxygen depletion especially towards the center of the spheroids [42], [50], [55]. This indicates that oxygen transfer limitation is not specific for the PEG hydrogel matrix but is also likely to be influenced by the close cell-cell contact and high cell density in 3D cultures.

### Protein and mRNA levels of LNCaP markers in 3D cultures differ from 2D cultures upon stimulation with 1 nM R1881 for 48 h

**Figure 4:**
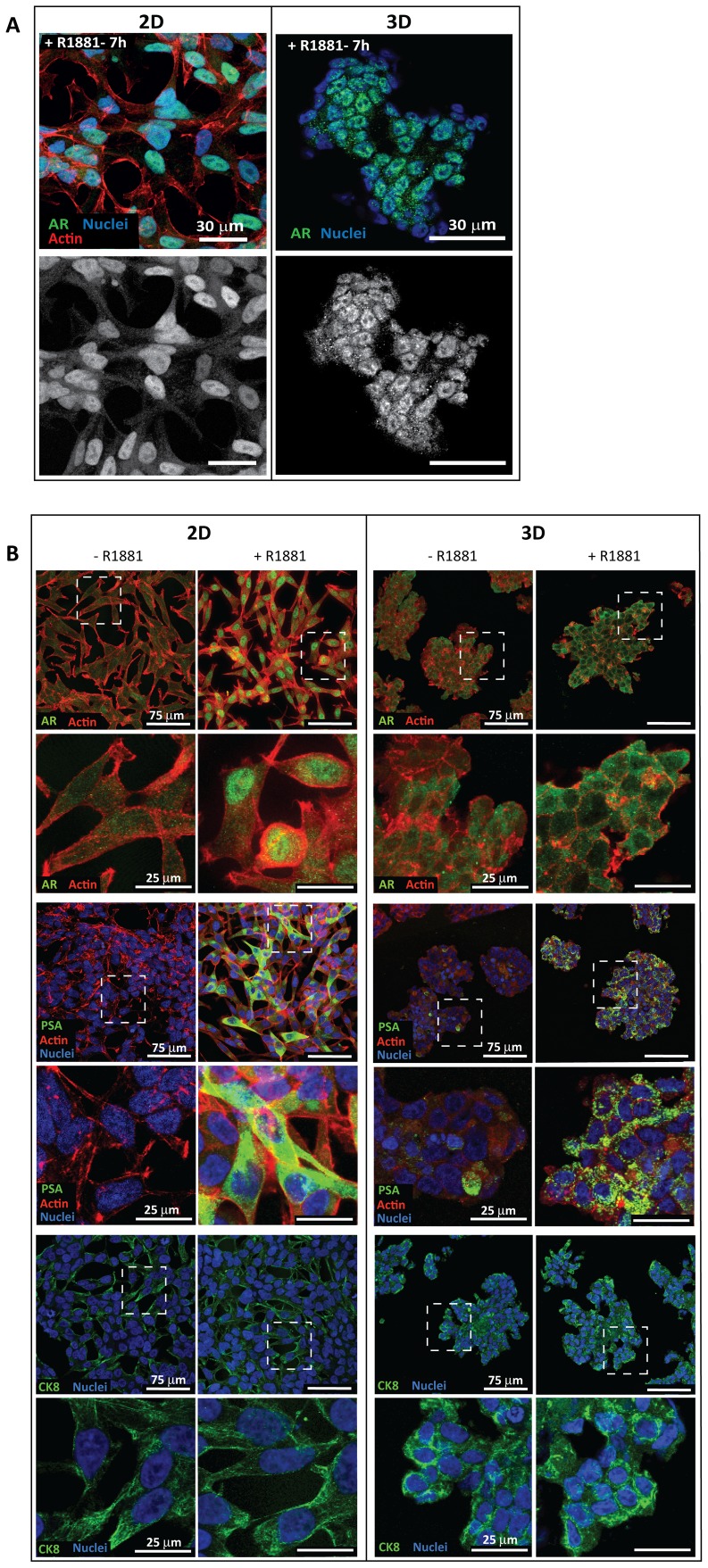
Phenotypic comparison of LNCaP cells in response to 1 nM R1881 in 2D and 3D cultures. (A) Immunofluorescent stainings of LNCaP cells after 7 h treatment with R1881 show co-localization of the AR and cell nucleus in both 2D (day 6) and 3D (day 28) cultures (top panel). Corresponding AR localization in both cultures is shown in grayscale images (bottom panel). (B) The cell phenotypes of 2D (day 6, left panel) and 3D (day 28, right panel) cultures grown in androgen depleted media or R1881 supplemented media (48 h) were compared by immunofluorescent stainings. Proteins of interest are in green, actin in red and nucleus in blue. In 2D cultures, AR is localized in the nucleus and PSA in the cytoplasm upon R1881 stimulation. Without treatment, the AR remains in the cytoplasm and PSA is not detected. The luminal epithelial marker, CK8 is detected in both cultures regardless of treatment condition. In 2D cultures, CK8 clearly stains the filament fibers while in 3D, CK8 is localized at the cell border. In 3D cultures, extranuclear AR localization is observed in both non treated and R1881 treated multicellular aggregates. PSA is also produced abundantly in R1881 treated 3D cultures but only at a very low level when not treated. Magnified regions of each specific staining and culture condition (white boxes) are shown below the corresponding images. CLSM images were taken at 60× magnification (1.4 NA) for A and 40× magnification (1.25 NA) for B. Scale bars: (A) 30 µm, (B) 75 µm and 25 µm (magnified regions).

During the initial stage of CaP, the large majority of tumors are androgen dependent and responsive; furthermore, the AR signaling pathway plays an important role in tumor development and is important throughout progression [56]–[57]. For this reason, androgen dependent LNCaP cells are widely used in 2D cultures for studying androgen receptor (AR) signaling, which is activated by androgens and analogues such as R1881. In order to expand on our knowledge of the LNCaP response to R1881 in 2D culture based experiments, we investigated the effects of R1881 on CaP marker expression in 3D cultures (Figure 4). Immunofluorescent staining revealed translocation of AR into the cell nucleus in both 2D and 3D cultures after 7 h of 1 nM R1881 treatment (Figure 4A). It is well established in 2D culture that once AR binds to R1881, it is transported to the nucleus where it initiates transcription of target genes, such Prostate Specific Antigen/Kallikrein 3 (PSA) [58]. Our preliminary study indicated that AR target genes, such as PSA, were induced at higher levels after prolonged R1881 treatment (after 36 h) in both 2D and 3D cultures (Figure S5A). Hence, we chose for this study a 48 h R1881 treatment for both cultures. Interestingly, though 2D and 3D cultures responded to the 48 h R1881 treatment, as evidenced by an increase in the mRNA and protein levels of PSA, localization of AR in cell nucleus was detected to a lesser extent in 3D cultures compared to 2D cultures. However, neither localization nor intensity of the luminal epithelial marker, Cytokeratin 8 (CK8) was affected by R1881 treatment in 2D or 3D cultures.

**Figure 5:**
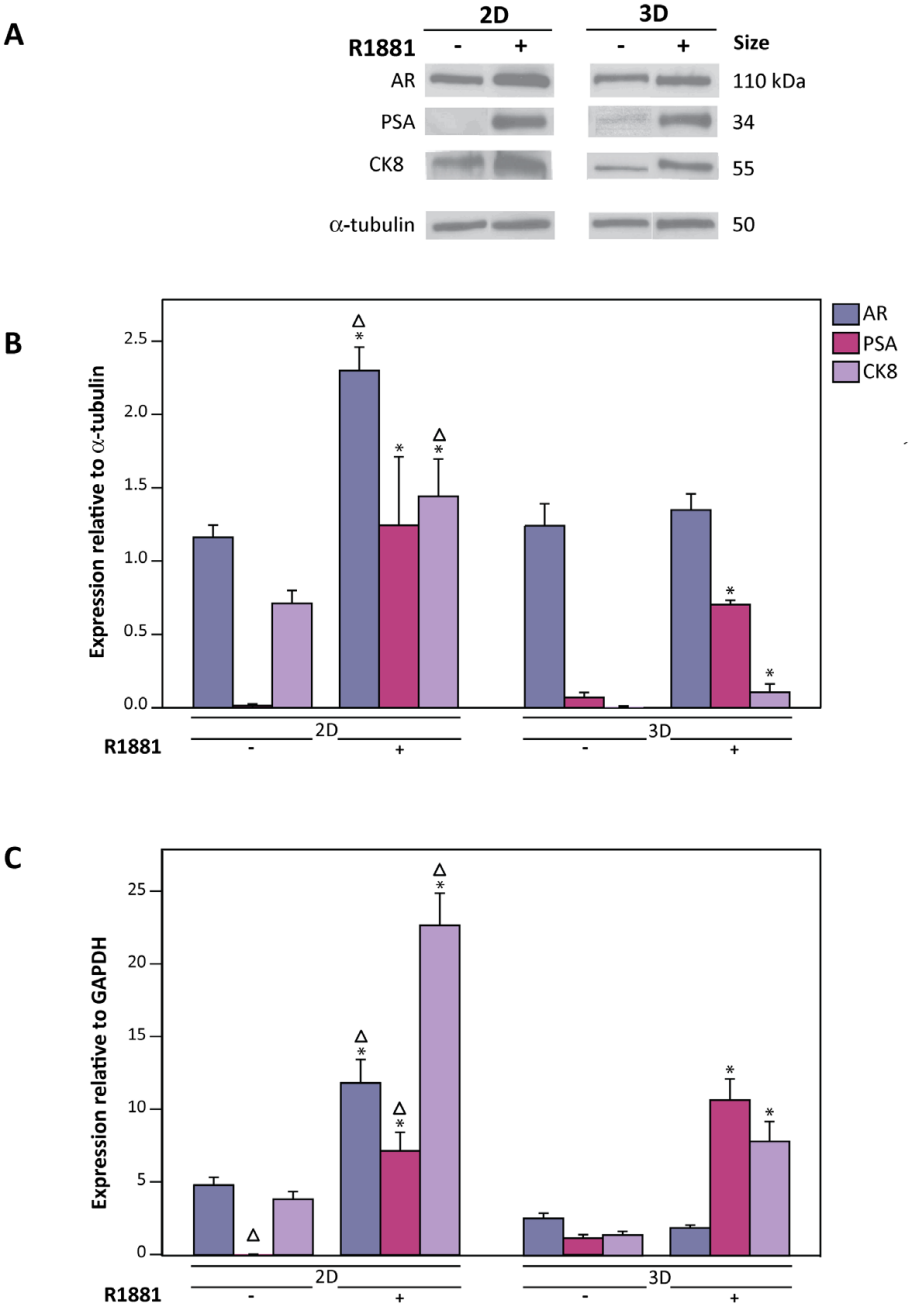
Protein and mRNA levels of 2D and 3D cultures in response to 1 nM R1881 after 48 h of treatment. (A) A representative Western blot reveals an increased level of all the investigated proteins in 2D cultures when treated with R1881 compared to the non treated (ethanol controls) while in 3D cultures, only PSA and CK8 are significantly elevated. (B) Signal ratio of proteins relative to α-tubulin from Western blots of R1881 treated and ethanol control groups (−R1881) are presented as mean ± SE. The protein quantifications support the changes shown in immunoblots above. (C) qRT-PCR representing expression of LNCaP cell markers (mean ± SE) where PSA and CK8 are up-regulated at the mRNA level upon treatment in both 2D and 3D cultures when compared to non treated 2D or 3D groups. The AR is only elevated in 2D cultures. Significant difference (p<0.05) between treated and non-treated samples within similar groups (either 2D or 3D cultures) is denoted by (*) and between similar treatment of different groups are denoted by (Δ). Triplicate samples were analyzed from at least three independent experiments.

To examine the effect of R1881 on levels of proteins, we focused on the expression of the AR, PSA and CK8 by performing Western blot analysis. Upon R1881 treatment, PSA and CK8 levels were enhanced in 2D and 3D cultures compared to non treated ethanol controls although elevation of CK8 protein by R1881 was not apparent in immunofluorescence staining in 2D and 3D cultures, which may be attributed to a lack of the sensitivity (of the antibody used) (Figure 5A, B). Interestingly, in treated 2D cultures, AR protein levels were elevated when compared to non-treated controls, indicative of stabilization of ligand bound AR and/or an increase in AR production [59]–[60]. In contrast, no significant changes were detected in 3D cultures. Taken together, this suggests a differential regulation of AR protein expression in 2D and 3D cultures in response to R1881. The effect of R1881 on mRNA levels in 2D and 3D cultures was further investigated by quantitative Real Time-Polymerase Chain Reaction (qRT-PCR). In accordance with the increased protein levels, PSA and CK8 mRNA levels increased in both cultures (Figure 5C). Similar to western blot analysis, the AR mRNA level was not significantly affected by R1881 treatment in 3D cultures but did show a declining trend when treatment was prolonged (Figure S5B). Despite higher AR expression in 2D cultures compared to 3D cultures, PSA expression was similar between cultures, as shown in Figure 5C. Intriguingly, in non treated cultures, the baseline PSA mRNA level was significantly higher in the 3D relative to 2D cultures. AR and PSA mRNA levels in LNCaP tumor xenografts from intact Non obese diabetic/Severe Combined ImmunoDefficiency (NOD/SCID) mice (Figure S5C) were comparable to the R1881 stimulated 3D cultures but not the 2D cultures. Thus, our findings indicate that our 3D cultures better reflect *in vivo* tumors.

### Androgenic response of LNCaP cells in 3D cultures is altered compared to 2D cultures under an R1881 deprived condition

**Figure 6:**
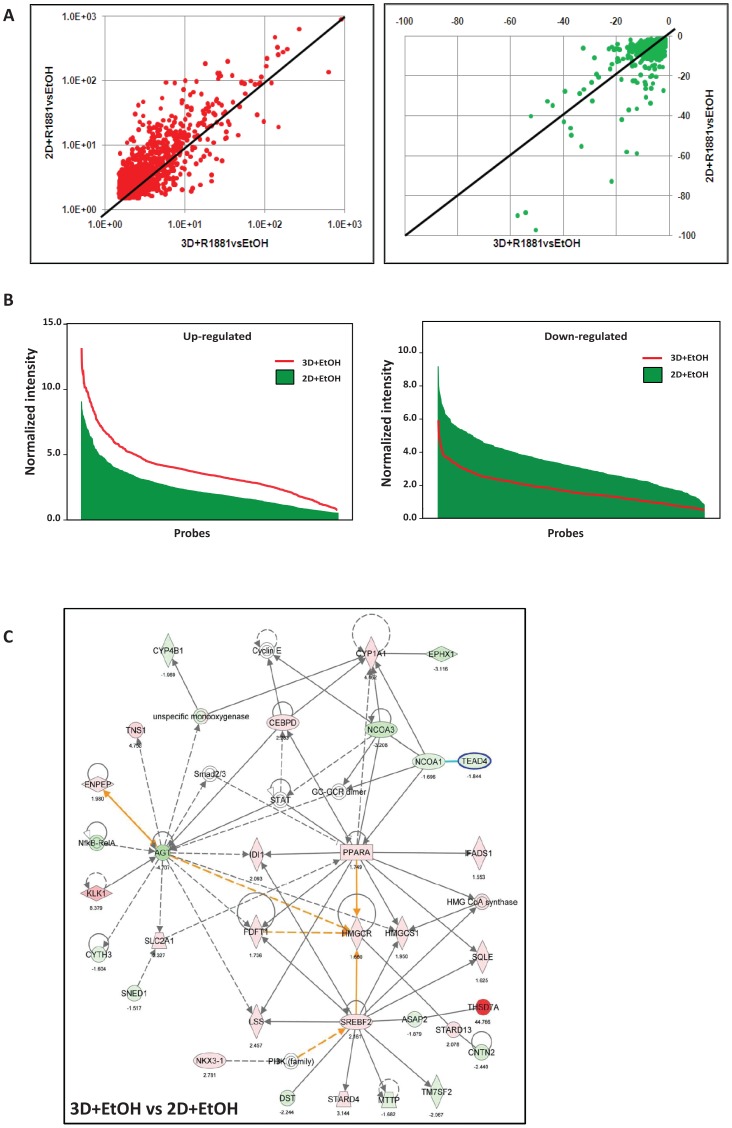
Microarray expression profile of androgen regulated genes in 2D and 3D LNCaP cell cultures. (A) Comparison of the fold change in expression levels of 1165 up-regulated (red) and 1697 down-regulated androgen-responsive genes (green) in LNCaP 2D and 3D cultures treated with 1 nM R1881 for 48 h relative to the corresponding EtOH controls. There is an increased magnitude of fold change in 2D cultures compared to 3D cultures upon R1881 treatment as represented by the population of red dots above the linear curve (left) and green dots below the linear curve (right). (B) Normalized intensity of probes representing 1469 differentially expressed genes that were up-regulated (left) and down-regulated (right) by androgen was compared between 2D+EtOH and 3D+EtOH cultures. The base line level is higher in 3D for the androgen up-regulated genes but lower in the androgen down-regulated genes when compared to 2D cultures. (C) Ingenuity Pathway Analysis (IPA) of the 1180 commonly regulated androgen-responsive genes. The network shows genes involved in lipid and steroid metabolism. Unbroken lines indicate direct interactions and broken lines indicate indirect interactions. The numbers and colors represent the fold change in expression levels (green = down-regulated and red = up-regulated) observed in the comparison of the ethanol controls of the 2D and 3D cultures (3D+EtOHvs2D+EtOH).

Given the above differences in PSA and AR expression and AR localization between 2D and 3D LNCaP cell cultures, we performed microarray analysis to comprehensively assess transcriptional differences between 2D and 3D LNCaP cell cultures under androgen deprived and R1881 treated conditions, focusing on androgen-responsive genes. We found differential regulation of 4157 (1896 up, 2261 down) and 3306 (1304 up, 2002 down) androgen-responsive genes in R1881-treated 2D and 3D LNCaP cell culture, respectively. The R1881-treated 2D (2D+R1881) and 3D (3D+R1881) cultures shared 2862 commonly regulated androgen-responsive genes (1165 up- and 1697 down-regulated genes) when compared to the respective ethanol controls (Figure 6A). Interestingly, the fold change in expression level of 898 (31%) of these genes were substantially reduced in 3D+R1881 when compared to 2D+R1881. This was particularly evident for up-regulated genes, as shown in the scatter plots (Fig. 6A), suggesting an androgenic response in the absence of androgens when the LNCaP cells were grown in the 3D culture. Comparison of the 3D and 2D ethanol controls (3D+EtOHvs2D+EtOH) revealed that 1469 androgen-responsive genes were differentially expressed (Figure 6B). This strongly suggests that decreased fold change of androgen regulated genes in response to R1881 in the 3D culture relative to 2D+R1881 was caused by a strong shift in the base line expression level of the androgen regulated genes (Fig. 6B). The difference in base line expression of probes representing the 1469 androgen regulated genes between 3D and 2D was evident in both androgen up or down-regulated genes. This is further supported by our qRT-PCR results that revealed a significantly higher transcription level of androgen regulated genes, such as PSA (Figure 5C), Kallikrein 2 and Kallikrein 4 (Figure S6), in untreated 3D cultures compared to untreated 2D cultures. We then examined 1180 commonly regulated androgen-responsive genes (483 up- and 697 down-regulated genes) by pathway analysis with Ingenuity Pathway Analysis (IPA) software. In support of the strong androgenic response triggered by growing LNCaP cells in 3D culture, the IPA analysis ranked biosynthesis of steroids as the top canonical pathway. This ranking was based on the differential regulation of genes like farnesyl-diphosphate farnesyltransferase 1 a (FDFT1), 3-hydroxy-3-methylglutaryl-CoA reductase (HMGCR), isopentenyl-diphosphate delta isomerase 1 (IDI1), lanosterol synthase (LSS) and squalene epoxidase (SQLE) (results not shown). Furthermore, the top networks listed by the IPA analysis were associated with lipid and steroid metabolism (Figure 6C). They contain node molecules like angiotensinogen (AGT), peroxisome proliferative activated receptor alpha (PPARA) and sterol regulatory element binding transcription factor 2 (SREBF2). Table 1 illustrates the fold change in expression levels of a selection of classical androgen regulated genes and members of the lipid and steroid biosynthesis network in response to R1881 and culture conditions (2D and 3D). We have previously shown that LNCaP cells were capable of *de novo* steroidogenesis, and that these pathways can be further induced by external signals such as insulin [61]–[62]. Altogether, these results show that growing LNCaP cells in the absence of androgens in the 3D culture augmented molecular hallmarks of an androgenic response and activated pathways responsible for cholesterol and steroid biosynthesis. Future experiments will address if factors such as increased in cell density and facilitation of cell-cell communication through homeotypic cell contacts (Figure 2–4) are responsible for the activation of androgen regulated genes and/or if *de novo* steroidogenesis is increased in the 3D LNCaP cell culture.

**Table 1:** Fold changes in expression level of selected classical androgen regulated genes in 2D and 3D LNCaP cell cultures.

Gene	2D R1881vsEtOH	3D R1881vsEtOH	EtOH 3Dvs2D
Kallikrein 3 (KLK3)	60.19	11.65	17.44
Chemokine receptor 4 (CXCR4)	93.13	5.77	12.55
Kallikrein 2 (KLK2)	129.29	27.21	8.98
N-myc downstream-regulated gene (NDRG) family member 1 (NDRG1)	18.79	4.33	6.57
Transmembrane protease, serine 2 (TMPRSS2)	36.78	18.39	3.51
15-hydroxyprostaglandin dehydrogenase (HPGD)	183.78	18.03	3.15
Homeobox protein Nkx-3.1 (NKX3-1)	11.13	4.62	2.78
FK506 binding protein 5 (FKBP5)	90.43	55.73	1.74
17-beta-hydroxysteroid dehydrogenase type 2 (HSD17B2)	47.09	2.99	1.77
StAR-related lipid transfer (START) domain containing 4 (STARD4)	6.79	2.41	3.14
Lanosterol synthase (LSS)	10.49	3.82	2.46
Sterol regulatory element binding transcription factor 2 (SREBF2)	2.57	2.69	2.16
3-hydroxy-3-methylglutaryl-CoA synthase 1 (HMGCS1)	13.19	3.87	1.95
Peroxisome proliferator-activated receptor alpha (PPARA)	2.44	1.55	1.75
Angiotensinogen (AGT)	−5.58	−1.64	−4.71

Expression level changes of classical androgen regulated genes (top) and genes of the lipid and steroid biosynthesis pathway (bottom) were derived from indicated comparisons between R1881 and ethanol (EtOH) treated 2D and 3D LNCaP cell cultures.

**Figure 7:**
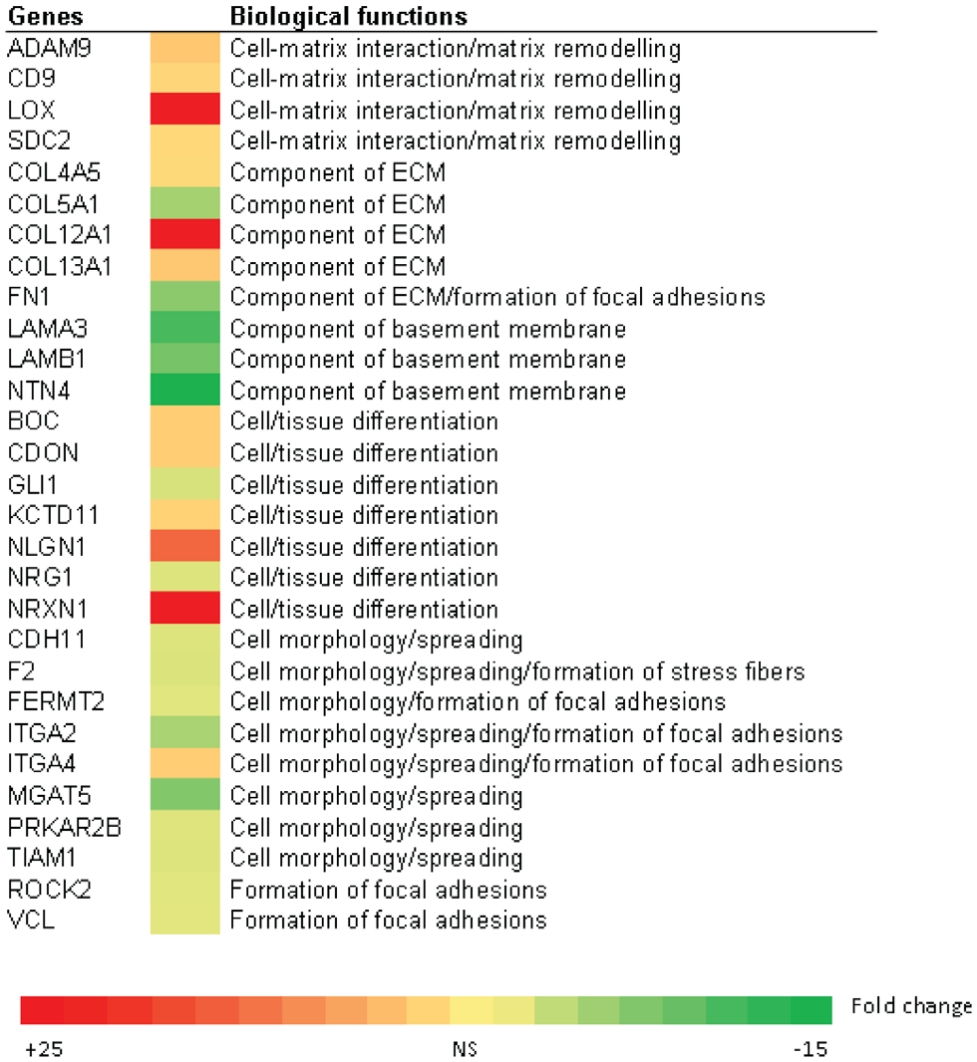
Heat map of gene expression level comparing untreated 3D cultures to 2D cultures (3D+EtOHvs2D+EtOH). The fold change in expression levels of genes related to cellular functions are represented by the colors in the heatmap. The green color denotes a decrease in expression in 3D cultures relative to 2D cultures and the red color denotes an increase in 3D cultures relative to 2D cultures. The fold change between −1.5 to +1.5 is considered not significant (NS). The differential expression of these molecules suggests that LNCaP cells grown in 3D may assume different roles in cell-cell signaling and interactions from the 2D cultures.

Apart from steroid biosynthesis, other top molecules highlighted in 3D culture by IPA analysis were associated with cell cycle, cellular movement, cell morphology and cell-to-cell signaling and interaction (Figure 7). In the 3D culture compared to 2D under uninduced condition, there was higher transcription of enzymes involved in matrix remodeling, particularly lysyl oxidase (LOX), as well as expression levels of genes encoding for ECM proteins (collagens, e.g. COL12A1). There was also a higher expression of genes such as neurogelin 1 (NLGN1) and neurexin 1 (NRXN1), suggesting a greater cell differentiation capacity in the 3D microenvironment. We also found lower expression levels of genes related to focal adhesion formation and actomyosin contractility, such as vinculin (VCL) and Rho-associated, coiled-coil containing protein kinase 2 (ROCK2) in 3D cultures may be also related to the lower stiffness experienced by the cells within the hydrogel compared to 2D. These molecular changes reflect the difference in cell morphology of 2D and 3D cultured cells as also reported by others [12], [63]–[66].

## Discussion

This study presents a phenotypic and genotypic comparison between LNCaP cells cultured within microenvironments engineered with specific functionalities to represent the tumor microenvironment and LNCaP cells grown on 2D tissue culture plastic. The acknowledgement of the role of the tumor cell microenvironment in modulating cell signaling, migration and differentiation amongst other cellular responses has led to the utilization of 3D *in vitro* cultures for cancer studies. Evidence from previous studies shows that 3D cultures are by far more physiologically representative of the cancer microenvironment than monolayer cultures. To date, the most common approaches used in *in vitro* to generate 3D compact aggregates (also sometimes known as spheroid), that approximate the *in vivo* tumors is by liquid-overlay and embedding cells in either natural or synthetic ECM hydrogels [55], [67]–[68]. By culturing the cells within the synthetic PEG hydrogels, this promotes a 3D cell-cell and cell-matrix interactions as oppose to the otherwise restricted geometry of 2D surface. While natural hydrogels are more commonly used for culturing cancer cells, the biophysical, biochemical and biological properties of natural hydrogels cannot be independently tailored. On the other hand, the synthetic hydrogel that allows better definition of its biochemical and biophysical properties is emerging as a potential substitute for the natural hydrogels in cancer research [24]. In this study, LNCaP cells were cultured within the synthetic PEG hydrogel functionalized with key biomimetic features of the natural ECM, specifically cell binding RGD motifs and MMP cleavage sequences. Addition of these peptides provides adhesion sites for cells and allows controlled matrix degradation by MMP activity [30], similar to the *in vivo* situation. Through characterization of the bioengineered microenvironment as well as the cell behavior, we have established and validated a 3D culture system that allows studying of some aspects of tumor-like morphogenesis and their response to hormone agonist stimulation.

Matrix properties, such as ligand composition and stiffness have been shown to influence properties which are associated with the malignancy of tumor cells, such as migration, morphology and proliferation [8]–[10], [69]. An *in vitro* study by Tilghman *et al* (2010) reported that the growth of multiple cancer cell lines is either dependent or not dependent on the matrix rigidity. This observation *in vitro* can help to predict the cell line's response to certain ECMs *in vivo* [10]. We also investigated if the LNCaP cells were sensitive to the change in matrix stiffness. The stiffness of these biomimetic hydrogel matrices can be easily tuned by altering their dry-mass content. This allowed us to profile the growth of cells cultured in the biomimetic hydrogels of three different stiffnesses with constant cell ligand densities. As anticipated, the increasing dry-mass content from 1.5 to 2,5% contributes to the matrix stiffness. Consistent with earlier studies which used different cell types cultured in the same synthetic hydrogels [30]–[31], we showed that matrix stiffness has a strong influence on LNCaP cell growth in 3D. While cells in softer matrix were able to proliferate faster, in stiffer matrix (2.5% PEG, 8 kPa), growth was impeded. We ruled out nutrient deficiency as the reason for non growth in 2.5% hydrogels, as our study had shown that molecules of molecular weight up to 66 kDa, a range that encompasses most growth factors, could penetrate the hydrogels. Moreover, cells close to the surface of the 2.5% hydrogel were not able to grow despite having full access to nutrients. Similarly, as shown earlier by Bott *et al.*(2010), human dermal fibroblasts in stiff hydrogels were viable but did not proliferate into multicellular aggregates such as normally observed in softer matrices [30]. Therefore, we conclude that the higher rigidity of the 2.5%, compared to 1.5 and 2% hydrogels, may be the cause of inhibition of LNCaP cell growth. The proliferation of LNCaP cells in 2% hydrogels is comparable to the growth of *in vivo* tumor and CaP xenografts, in terms of the growth pattern and rate [44]–[46], [70]–[71]. The 2% hydrogels are not only compatible for LNCaP cell growth in a manner that recapitulate the slow growing tumor but also resemble the stiffness of glandular tissues (4 kPa) and other soft tissues [5], [72].

In addition to the growth kinetics of LNCaP cells in the 3D cultures, morphological features of the LNCaP multicellular colonies show resemblance to *in vivo* tumors, such as compact cell-cell adhesion and the presence of hypoxia and apoptotic cores. Hypoxia and apoptotic cores are frequently detected and observed in numerous *in vitro* 3D models of LNCaP cells and other tumor cells, as well as *in vivo* tumors [17], [41]–[42], [50]–[51], [73]–[74]. In our study, presence of hypoxia and lack of nutrients could explain the growth kinetics in the 3D cultures. As the size of the colony size increased, nutrient and oxygen access to cells located at a greater radial distance from the colony periphery may decrease. This finding was exemplified by a greater Pimonidazole stained region in larger LNCaP colonies (day 28) compared to smaller colonies (day 14). As these aggregates reached a critical size, the nutrient deprived cells may become quiescent or undergo cell death, which may lead to apoptotic/necrotic core formations. Additionally, loss of matrix attachments experienced by cells in the centre may also lead to anoikis [75]. While apoptotic cores are not always apparent in vascularized *in vivo* tumors, heterogeneously distributed hypoxic regions are detected in these tissues [76]–[78]. Therefore, our 3D model which only allows growth of the LNCaP colonies up to a certain size or cell density is more representative of an early development of avascular/poorly vascularized tumors rather than a vascularized tumor. All these features that reflect characteristics of avascular tumors, however, are not present in 2D cultures.

Multiple ‘finger-like’ structures were detected in LNCaP colonies after 28 days of cultures. Similar ‘fingering’ phenomenon was also described by Anderson *et al.*(2006), where adaptive tumor cells in spheroids acquired the fingering structure under nutrient or oxygen depleted conditions [79]. This morphology is also associated with collective cell migration, which occur when cancer cells in aggregates invade 3D matrices [80]–[81]. This could also be related to the gain of invasive traits by cells in spheroids grown for a longer period of time [49], indicating that cells could become more invasive in a microenvironment with prolonged metabolite or mechanical stress. These stresses are also constantly exerted upon growing tumor cells and are believed to selectively favor survival of the more aggressive cell phenotype [79], [82].

Our data further indicated a differential kinetics in the cellular response to the synthetic androgen, R1881 among 2D and 3D cultures. Up-regulation of PSA was evident with 48 h treatment of R1881 in 2D and 3D cultures suggesting R1881-mediated activation of AR. Although the dynamics of the AR have been extensively explored, the AR machinery system is still not fully understood. Our study showed that, contrary to 2D cultures, the nuclear AR was greatly reduced in the 3D cultures after prolonged (48 h) treatment with R1881 but did not hinder induction of androgen target genes as also reported by Kesler *et al* (2007) [83]. However, distinct nuclear AR was observed at the early stage (7 h) of R1881 stimulation, indicating the AR import to the nucleus did occur and was not impaired.

Clearly, the kinetics of AR turnover within 48 h of R1881 stimulation in 3D cultures differs from 2D cultures while still maintaining the expression of androgen responsive gene, PSA. In the absence of R1881, expression of androgen responsive genes, such as KLK2 and 4, was higher in 3D cultures compared to 2D cultures despite the absence of R1881. Microarray gene analysis also showed that genes responsible for cholesterol and steroid biosynthesis were also elevated in 3D untreated cultures. We hypothesize that the increase in cell-cell interaction/cell density could elevate the baseline expression of the androgen regulated genes (unpublished data). Indeed, studies have shown that integrins and hypoxia can influence AR activity by altering the sensitivity of AR to androgens [84]–[89].

In conclusion, the bioengineered LNCaP tumors reflect *in vivo* tumors to more extensively than 2D cultures. Morphologically, they bear resemblances to avascular tumors, and phenotypically, like early androgen responsive CaP, they can be induced to respond to androgen in a manner similar to LNCaP xenografts. The discrepancies we described in the androgen response and AR transport between 2D and 3D cultures highlights the importance of the microenvironment and should be further investigated. Considering the role that AR and androgen play in CaP progression, we may need to further investigate the mechanisms of AR signaling in a 3D context. Therefore, we suggest that this 3D model will be of great value in extending our understanding of tumor cell biology and may be utilized in designing a more physiological *in vitro* drug screening platform, which may help to advance CaP clinical outcomes.

## Materials and Methods

### Materials

The synthetic androgen, R1881 was purchased from DuPont. Pimonidazole hydrochloride (Hypoxyprobe™-1) was used for detection of hypoxic cells as described previously [73]. Cells were incubated with 100 µm Pimonidazole hydrochloride in media for 4 h prior to the fixation process. For immunostaining and Western blotting, primary antibodies against E-cadherin (Invitrogen), Pimonidazole (Hypoxyprobe™-1), Caspase-8 (Abcam), androgen receptor C19 (AR, Santa Cruz), Cytokeratin 8 M20 (CK8, Abcam), and Prostate specific antigen (PSA, Dako cytomation) were used. Secondary antibodies for Western blotting are horseradishperoxidase (HRP)-conjugated goat anti-rabbit and HRP-conjugated rabbit anti-mouse. They were used at 1∶2000 dilutions. Secondary anti-mouse and anti-rabbit Alexa 488 antibodies at 1∶300 dilution (Molecular Probes) were used for immunofluorescent staining. For diffusion measurements, food dye, E133 and fluorescein isothiocyanate conjugated bovine serum albumin (FITC-BSA) kindly provided by Dr. Jonathan Harris were used as tracers.

### Cell culture

The LNCaP prostate cancer cell line (American Tissue Culture Collection, Rockville, MD) was cultured in RPMI (Invitrogen) supplemented with 10% FBS (Hyclone) and 1% penicillin-streptomycin (Invitrogen). LNCaP cells were obtained from passage 18 and were subcultured 2–8 times before experimentation. For all 2D experiments, 1×10^−4^ cells/cm^2^ were seeded on tissue culture plastic or glass cover slips.

### Preparation of biomimetic PEG-based hydrogels

PEG-based hydrogels were prepared as previously described by Ehbar *et al.* (2007) [29]–[30], [90]. In summary, PEG-Gln/PEG-MMP-Lys precursor stock solution (5% w/v) prepared as described by Bott *el al* (2010) and Loessner *et al* (2010) was diluted to the desired PEG concentration in Tris-Buffer (50 mM, pH 7.6) containing 50 µM RGD conjugate, and 50 mM calcium chloride [30]–[31]. Following the addition of 10 U/mL thrombin-activated factor XIII, the LNCaP cell suspension was added into the reaction mixture to yield a final cell density of 3.5×10^5^ cells/mL (Figure 1A), and hydrogel discs were formed by sandwiching 20 µL drops of the reaction mixture between two sterile glass slides (separated by 1.5 mm spacers) pre-coated with SigmaCote (Sigma). Hydrogels were allowed to polymerize at 37°C for 20–40 min in an incubator (Binder Gmbh, Germany) before being transferred to 24 well plates filled with growth medium. Medium was changed every 4 days.

### Mechanical testing of hydrogels

To measure the global stiffness of cell-free hydrogels of 1.5, 2.0 and 2.5% PEG content, unconfined compression tests were conducted using an Instron microtester (Instron). Measurements were performed at 30% strain with displacement of 0.45 mm/min, using a 5 N cell load. During compression, hydrogels were maintained at 37°C in a moist condition. The elastic (Young's) modulus was extracted from the linear region of the stress-strain curve (9–15%) according to the equation E = F/A/L/L_0_. F = force applied to sample, A = cross section area which the force is applied, L = the amount of change in hydrogel thickness, L_0_ = the original thickness of the hydrogel.

Local stiffness of the hydrogels was examined by atomic force microscopy (AFM) indentation measurements using a NanoWizard II from JPK Instruments (Berlin, Germany). Pyramidal-shaped cantilevers (MLCT, Veeco) were calibrated *in situ* using built-in procedures of the AFM software. Coverslips with hydrogel discs were mounted into the temperature-controlled chamber of the instrument (Petridishheater, JPK Instruments). The measurements were conducted at 37°C in PBS. To measure the elastic (Young's) modulus, E of the PEG hydrogels, the cantilever was approached at a speed of 2 µm/sec onto the hydrogel until a contact force of 0.8 nN was reached. From the recorded force versus tip-sample-separation curves (Figure 1C, left), the elastic modulus was extracted using procedures implemented into the JPK IP software. This procedure applied a Hertzian fit assuming a pyramidal indenter with half-angle-to-face of 17.5°, and a Poisson's ratio of 0.5. For each gel, the elastic modulus was measured on at least 50 different spots on the gel. At least four gels from independent preparations were analyzed for each PEG concentration.

### Diffusion measurements

Cell-free hydrogel reaction mixtures of different PEG contents were pipetted into a pasteur pipette of approximately 2 mm diameter and allowed to polymerize at room temperature for 20 min. Then, the food dye, E133 (792 Da) (1∶1000 dilution) or FITC-BSA (66 kDa) (1 mg/mL) was injected into the pasteur pipette to come in contact with the hydrogel before incubation at 37°C. After 110 min (E133) and 50 min (FITC- BSA), the hydrogel-tracer solution intersections were imaged using a Nikon Eclipse microscope equipped with a Nikon Digital camera DXM1200C (Coherent Scientific). From the grey scale images, an intensity profile was plotted over the region from the gel-tracer solution interface to the tracer-absent position using image J. The intensity profile was fitted to the following equation f(x) = a*erfc (x/2√Dt) using IGOR Pro (wavemetrics).

D = diffusion coefficient, a = intensity of the tracer, x = distance of measured tracer from the gel-tracer interface and t = duration of hydrogel exposure to the tracer.

### R1881 treatment for 2D and 3D cultures

Both 2D and 3D cultures were maintained in androgen-deprived media (RPMI media containing 5% charcoal stripped serum) for 48 h prior to androgen treatment. Androgen deprivation was initiated when cultures reached 70–80% confluency for 2D cultures and on day 24 for 3D cultures. Media were then changed to androgen- deprived media containing 1 nM R1881. The cultures were treated with R1881 for 48 hr before imaging or protein and RNA extraction. 2D and 3D cultures incubated for another 48 hr in androgen-deprived media in the absence of R1881 (with 0.008% ethanol) were used as controls.

### Proliferation assay for 2D and 3D cultures

For 2D cultures, cells were plated in 24 well plates and harvested daily from day 1 to day 7 by adding 200 µL of Proteinase K (0.5 mg/mL). The cell suspension was incubated at 56°C for 12 h and stored at −80°C before a Pico Green (Invitrogen) proliferation assay was performed as reported elsewhere [91]. For 3D cultures, hydrogels of different PEG concentrations was harvested at day 1, 7, 14, 21 and 28 and also subjected to digestion with 300 µL of Proteinase K for 16 hr prior to a Pico Green assay. The digested cell suspension mixture was diluted with phosphate buffered saline/ethylenediaminetetraacetic acid (PBS/EDTA) buffer to allow readout within the range of the standard curve between 10 ng/mL and 2 µg/mL DNA. All samples were assayed in triplicate from three biological samples

### Live-dead staining

At day 24 of 3D cultures, hydrogels with cells were washed with PBS and incubated in 2 µg/mL fluorescein diacetate (FDA) solution diluted in PBS for 30 min at 37°C. Cells were then incubated for another 5 min in 20 µg/mL propidium iodide (PI) solution before washing thoroughly with PBS. Hydrogels were immediately analysed using a Leica SP5 confocal microscope (Leica). Z stacks over a range of 150 µm were imaged and 3D projections were created using the Leica Image Processing software.

### Phalloidin-DAPI staining and spheroid size and shape characterisation

Cells were fixed in 4% formaldehyde/PBS, permeabilised in 0.2% Triton X-100 and stained with 0.8 U/ml rhodamine conjugated Phalloidin (Invitrogen) and 2 µg/ml 4′,6-diamidino-2-phenylindole (DAPI) [Invitrogen] for 40 min as described [92]. Fluorescent confocal images stacks over a range of 100–200 µm were captured with Leica SP5 laser scanning confocal microscopy (CLSM). From the 3D projections, the size and shape factor of the spheroid were measured using Image J software.

### Immuno/fluorescent staining

LNCaP cells were either cultured in normal growth media or treated with R1881 as described previously. For 2D cultures, LNCaP cells were cultured on glass cover slips. 2D and 3D cultures were fixed with 4% paraformaldehyde/PBS for 30 min. Hydrogels from 3D cultures were either stored in 4°C or processed for cryosection. Hydrogels for cryosection were prepared by immersing them in OCT Tissue Teck/PBS (1∶1) solution for 45 min followed by another 45 min in OCT Tissue Tek (ProSciTech Pty Ltd). Then, the hydrogels were loaded into cryomoulds, fully immersed in OCT Tissue Tek and frozen with liquid nitrogen. Hydrogels were stored at −80°C until ready for sectioning. 7 µm thick sections were permeabilized with 0.2% Triton-X for 10 min and blocked for 1 hr with 1% BSA solution before incubation in primary (Ecad, 1∶100, Pimonidazole, 1∶100, AR, 1∶200, PSA, 1∶200 and CK8, 1∶200) and fluorescently conjugated secondary antibodies. Samples were counterstained with Phallodin and DAPI for 40 min. Images were captured using Leica SP5 CLSM (Leica).

### Cell lysate preparation and Western blotting assays

2D and 3D cultures of treated and non treated groups were harvested for lysate preparation in lysis buffer (1% Triton X-100, 150 mM NaCl, 10 mM Tris/HCl, pH 7.5, 1 mM EDTA and 25 mM NaF) containing protease inhibitor cocktail (Roche). For 3D cultures, hydrogels were dispersed using a pipette to release the embedded spheroids. 20 µg of protein was loaded onto a 10% sodium dodecyl sulphate (SDS) polyacrylamide gel and separated by electrophoresis for 2 h at 120 V. Proteins were transferred to a nitrocellulose membrane by wet transfer for 1 h at 100 V. After primary antibodies (AR, 1∶2000, PSA, 1∶3000 and CK8,1∶1000) and secondary HRP conjugated secondary antibody incubation, chemiluminescent Pierce ECL Western Blotting Substrate (Thermo Scientific) was added and membranes were exposed on X-ray films.

### RNA isolation and quantitative Real Time-PCR (qRT-PCR)

RNA extraction was performed with Trizol (Invitrogen) according to the manufacturer's instruction. For 3D cultures, three hydrogels were pooled before adding Trizol reagent. RNA concentrations were quantified using a Nanodrop-1000 (ND-1000). Samples with a 260/280 ratio higher than 1.7 were used for subsequent procedures. The samples were then treated with DNAse Amp grade I and reverse-transcribed using the cDNA synthesis method for the qPCR kit (Invitrogen). QRT-PCR was performed using the 7900HT Fast Real-Time PCR System (Applied Biosystems) and data analyzed with SDS2.3 software as described previously [92]. The sequences of all primers are as follows:

GAPDH, 5′-GCAAATTCCATGGCACCGT-3′ and

5′- TCGCCCCACTTGATTTTGG-3′,

PSA, 5′-AGTGCGAGAAGCATTCCCAAC-3′ and

5′- CCAGCAAGATCACGCTTTTGTT-3′,

AR, 5′- CTGGACACGACAACAACCAG-3′ and

5′- CAGATCAGGGGCGAAGTAGA-3′, and

CK8, 5- CTGGGATGCAGAACATGAGTATTC-3′ and

5′- GTAGCTGAGGCCGGGGCTTGT-3′.

### Microarray gene expression profiling

Triplicates of each condition were prepared for microarray profiling which was performed on a custom Agilent 4×180 k oligo array. This microarray incorporates Agilent human gene expression protein-coding probes as well as non-coding probes; with the probes targeting exonic regions, 3′UTRs, 5′UTRs, as well as intronic and intergenic regions. RNA was isolated with Trizol, followed by clean-up using a RNeasy Mini Kit (Qiagen) and DNAse on column treatment according to the manufacturer's protocol. RNA samples were analyzed by a Bioanalyzer (Agilent) to ensure the RNA was of high quality. 200 ng of RNA from each group was amplified and labeled according to the protocol for One-Color Microarray-Based Gene Expression Analysis (Low Input Quick Amp Labeling Kit, Agilent). The input RNA was reversed transcribed into cDNA, using an oligo-dT-promoter primer which introduces a T7 promoter region. The subsequent *in vitro* transcription uses a T7 RNA polymerase, which simultaneously amplifies target material and incorporates cyanine 3-labeled CTP. cDNA synthesis and *in vitro* transcription were performed at 40°C for 2 h, respectively. The labeled cRNA was purified with Qiagen's RNEasy mini spin columns and quantified using a Nanodrop-1000. 1650 ng cRNA from each sample were loaded onto 4×180 k custom microarray which contains the Agilent 44 k probe set (design number 014850) and allowed to hybridize at 65°C for 17 h. The arrays were scanned with the Agilent Microarray Scanner G2565CA.

### Microarray data analysis

The microarray data were processed with Agilent Feature Extraction Software (v10.7). A quantile between array normalization was applied and differential expression was determined using a Baysian adjusted t-statistic from a Linear Models for Microarray Data (LIMMA) linear model. The p-values were corrected for a false discovery rate of 5%. Normalized gene expression data of the experiment are Minimum Information About a Microarray Experiment (MIAME) compliant and have been submitted to Gene Expression Omnibus (GEO) with the accession number GSE33610. The gene expression levels are presented as log_2_ and were compared between two groups with a t-test. Genes that were significantly different between two groups were identified with a p value of < = 0.05, and an average fold change of > = 1.5. The androgen-responsive gene filter was generated from 4×180 k microarray data of DHT- and R1881-treated LNCaP 2D cell cultures and consists of 6598 genes commonly regulated by both androgens (Lehman M and Nelson CC, unpublished results). The microarray data from the 2D and 3D cultures were processed with the androgen-responsive gene filter to compare their regulation in response to treatment (R1881 versus ethanol) and culture conditions (2D versus 3D). The filtered gene lists were examined by Ingenuity Pathway Analysis (IPA, Ingenuity Systems Inc.) for functional annotation and gene network analysis.

### Statistical analysis

Univariate ANOVA post hoc Least Significant Difference (LSD) tests were used to determine the statistical significance of data between more than two conditions for hydrogel stiffness, diffusivity, spheroid size and shape factor measurements, qRT-PCR and Western blot.

## Supporting Information

Figure S1 **Mechanical properties of 2% PEG-based biomimetic hydrogels after culture with LNCaP cells.** (A) A small variation of the hydrogel's stiffness was detected between day 1 to day 54 cultures. (B) The volume of hydrogels remains unchanged from day 1 to day 28. Each grid box represents 1 mm×1 mm.
(TIF)

Figure S2 **3D confocal image projections of 1.5–2.5% PEG hydrogels after 24 h immersion in 1 mg/mL FITC-BSA- solution.** Images of the cut surface were taken to examine the penetration of the FITC-BSA- (66 kDa) across the thickness (1.5 mm) of the hydrogel discs (green). It shows that BSA-FITC can still saturate the 2.5% PEG hydrogel. Dotted lines demarcate the border of the hydrogels. Scale bars: 100 µm.
(TIF)

Figure S3 **3D confocal image projections of the of LNCaP cells grown in the PEG hydrogel and Matrigel™.** LNCaP cells were embedded in the PEG hydrogels as single cells (left) and allowed to be cultured up to 28 days. Cells cultured within Matrigel form spheroids with well defined shape similar to cells grown in PEG hydrogels (right). CLSM images were taken at 20× magnification (0.7 NA) for PEG hydrogel and 40× (1.25 NA) for Matrigel™. Scale bars: 100 µm.
(TIF)

Figure S4 **Immunofluorescent staining of LNCaP cells grown in the 2% PEG-based hydrogels for 14 days.** Cultures were pretreated with 100 µm Pimonidazole hydrochloride for 4 h before being harvested and fixed for cryosectioning. Pimonidazole (green) is detected in a small region of the LNCaP colony indicating presence of hypoxic cells. CLSM images were taken at 40× magnification and 1.25 NA. Scale bar: 30 µm.
(TIF)

Figure S5 **qRT-PCR of LNCaP cells treated with 1 nM R1881 in 2D and 3D cultures, and LNCaP xenograft.** (A) mRNA level of PSA in 2D and 3D cultures at different treatment durations shows maximum induction for 3D cultures at 36–48 h and 48–72 h for 2D cultures. (B) mRNA level of AR changes with the treatment duration in both cultures. The expression decreased at 36 h of R1881 treatment in 2D cultures compared to non-treated (ethanol) controls but increased thereafter. In 3D cultures, a slight suppression was observed as the treatment was prolonged for more than 48 h. (C) The LNCaP xenograft from (after 12 weeks of inoculation) intact NOD/SCID mice shows comparable AR and PSA expression levels to 3D cultures treated with 1 nM R1881 for 48 h.
(TIF)

Figure S6 **qRT-PCR of LNCaP cells under androgen deprived condition in 2D and 3D cultures.** LNCaP cells were cultured in RPMI+5% CSS for 48 h followed by another 48 h in RPMI+5% CSS+ EtOH (0.08%) before being harvested for RNA isolation. Expression of Kallikrein 2 and Kallikrein 4 are both significantly higher in 3D compared to 2D cultures.
(TIF)

Video S1 **Movie of LNCaP cells embedded in a 2% PEG-based biomimetic hydrogel at day 7 of culture.** The video was taken with widefield microscopy from day 7 to day 12. This shows that spheroids are formed by cell division within the spheroid and not by aggregation of neighboring cells. No active cell migration was observed in the culture.
(WMV)
