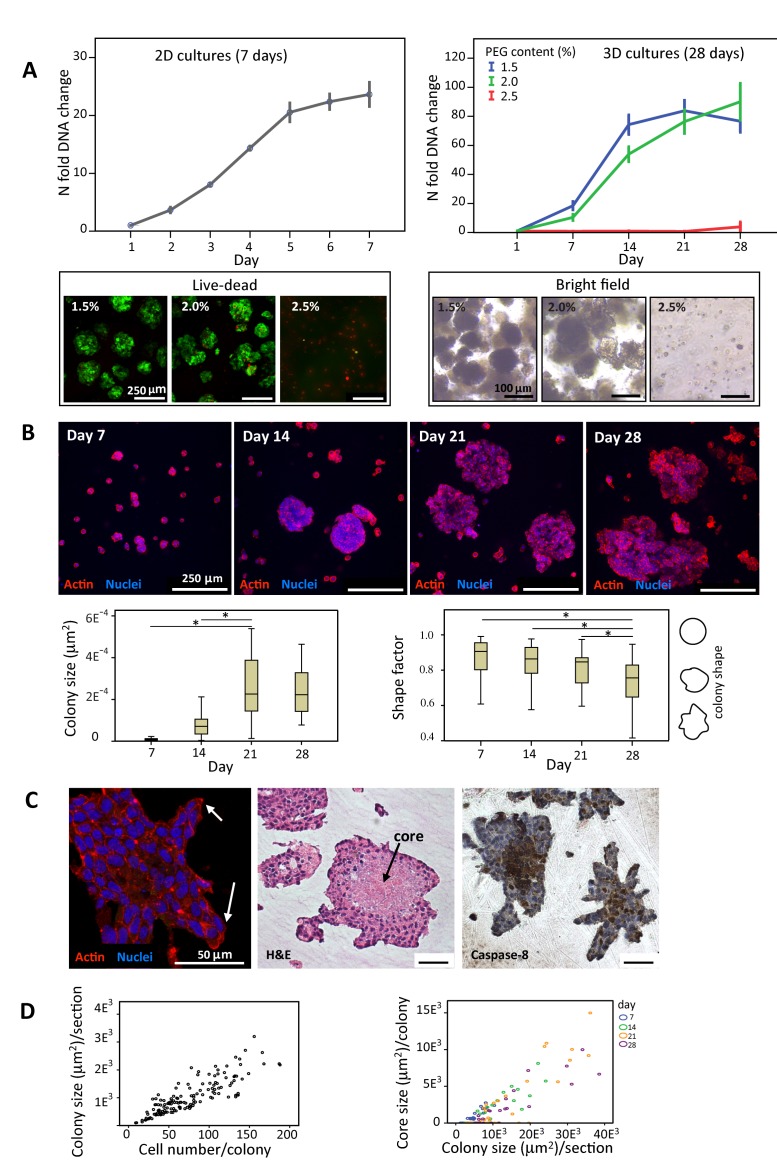# Correction: Phenotypic Characterization of Prostate Cancer LNCaP Cells Cultured within a Bioengineered Microenvironment

**DOI:** 10.1371/annotation/a26f1a2e-2765-4c10-942e-d2a738b80bf9

**Published:** 2013-01-17

**Authors:** Shirly Sieh, Anna V. Taubenberger, Simone C. Rizzi, Martin Sadowski, Melanie L. Lehman, Anja Rockstroh, Jiyuan An, Judith A. Clements, Colleen C. Nelson, Dietmar W. Hutmacher

The image incorrectly displayed as Figure 2 is a copy of Supplementary Figure S2. A correct version of Figure 2 can be seen here: 

**Figure:**