# Supplementary figures and images for: Phenotypic Characterization of Prostate Cancer LNCaP Cells Cultured within a Bioengineered Microenvironment
Source: PLoS One. 2012 Sep 5;7(9):e40217. doi: 10.1371/journal.pone.0040217 (PMC3434144; doi:10.1371/journal.pone.0040217)

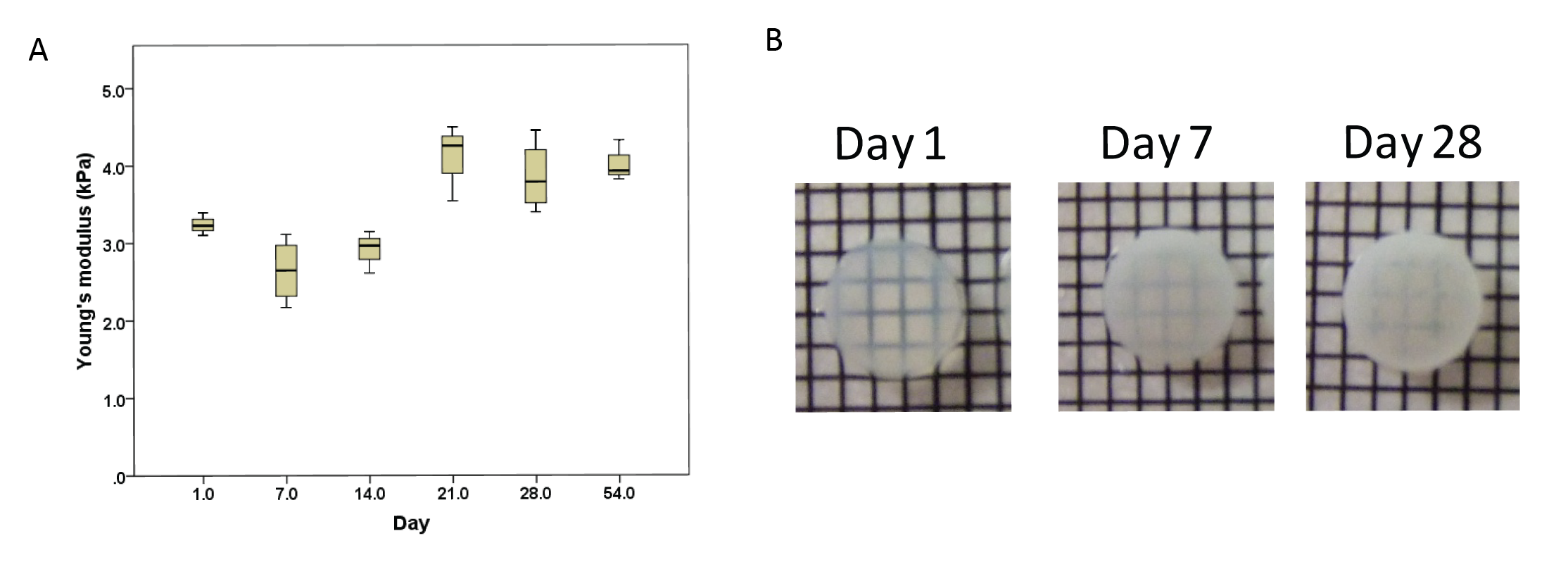

Supplement: Figure S1 — Mechanical properties of 2% PEG-based biomimetic hydrogels after culture with LNCaP cells. (A) A small variation of the hydrogel's stiffness was detected between day 1 to day 54 cultures. (B) The volume of hydrogels remains unchanged from day 1 to day 28. Each grid box represents 1 mm×1 mm. (TIF) [file pone.0040217.s001.tif]

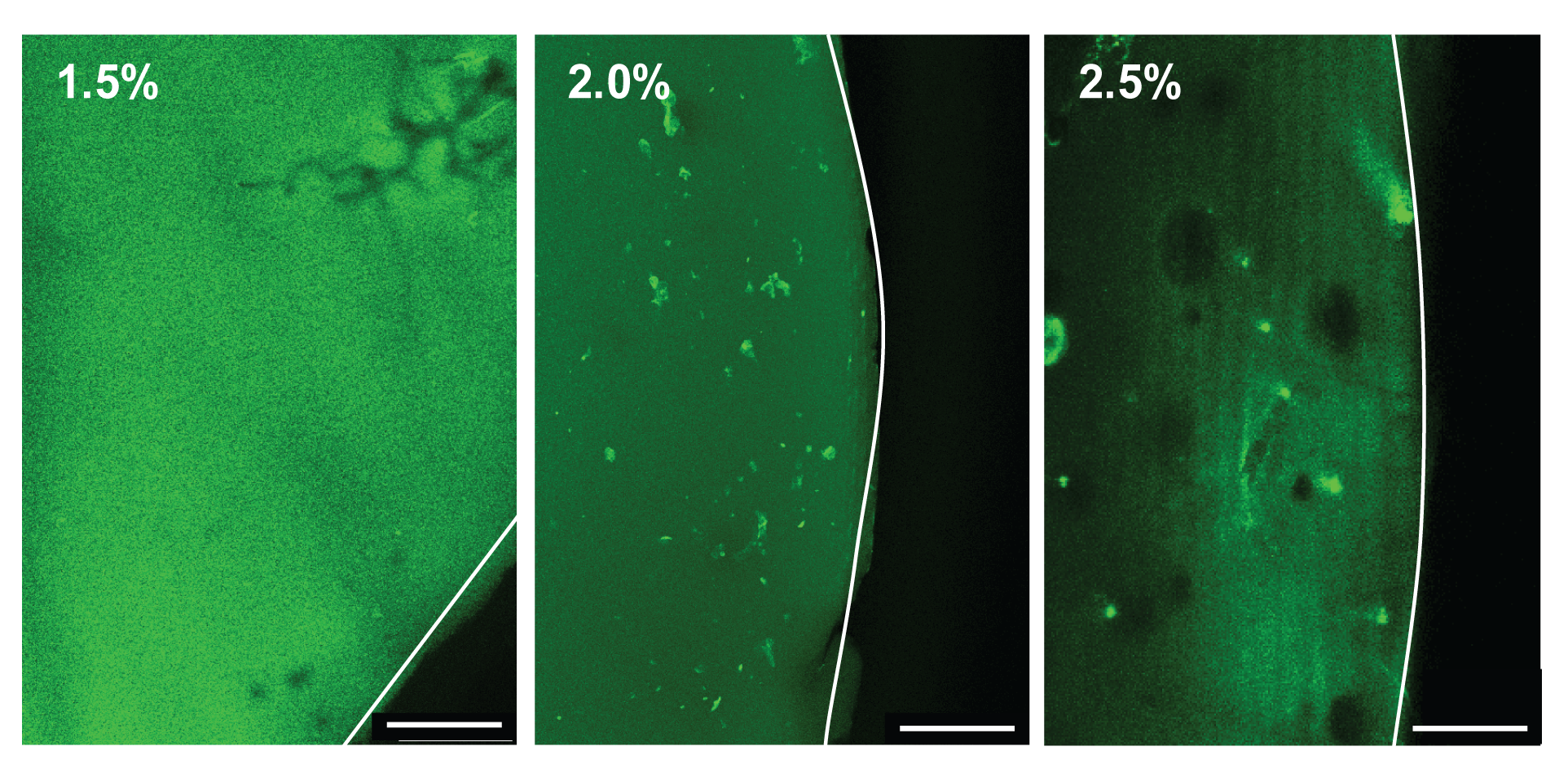

Supplement: Figure S2 — 3D confocal image projections of 1.5–2.5% PEG hydrogels after 24 h immersion in 1 mg/mL FITC-BSA- solution. Images of the cut surface were taken to examine the penetration of the FITC-BSA- (66 kDa) across the thickness (1.5 mm) of the hydrogel discs (green). It shows that BSA-FITC can still saturate the 2.5% PEG hydrogel. Dotted lines demarcate the border of the hydrogels. Scale bars: 100 µm. (TIF) [file pone.0040217.s002.tif]

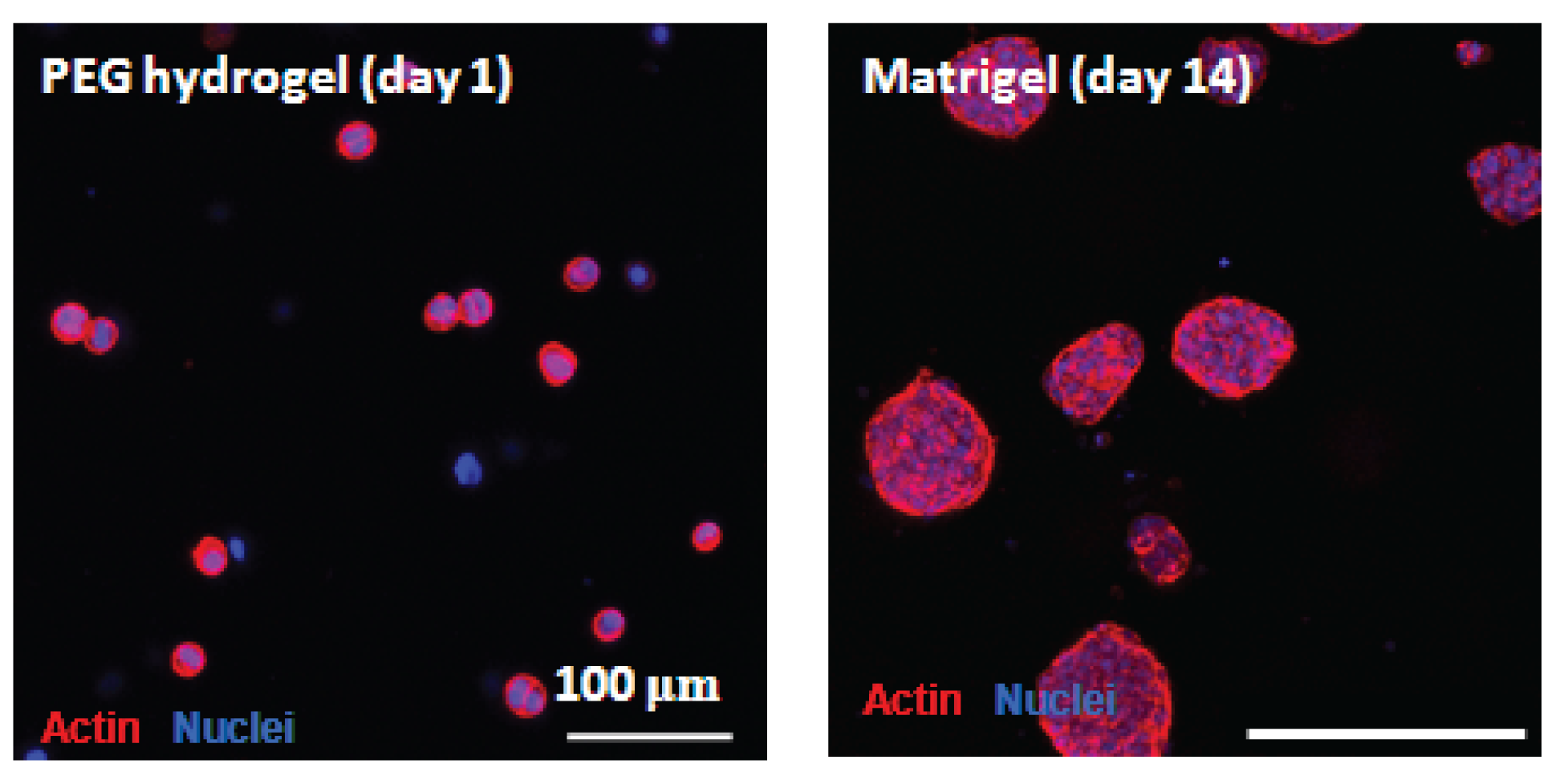

Supplement: Figure S3 — 3D confocal image projections of the of LNCaP cells grown in the PEG hydrogel and Matrigel™. LNCaP cells were embedded in the PEG hydrogels as single cells (left) and allowed to be cultured up to 28 days. Cells cultured within Matrigel form spheroids with well defined shape similar to cells grown in PEG hydrogels (right). CLSM images were taken at 20× magnification (0.7 NA) for PEG hydrogel and 40× (1.25 NA) for Matrigel™. Scale bars: 100 µm. (TIF) [file pone.0040217.s003.tif]

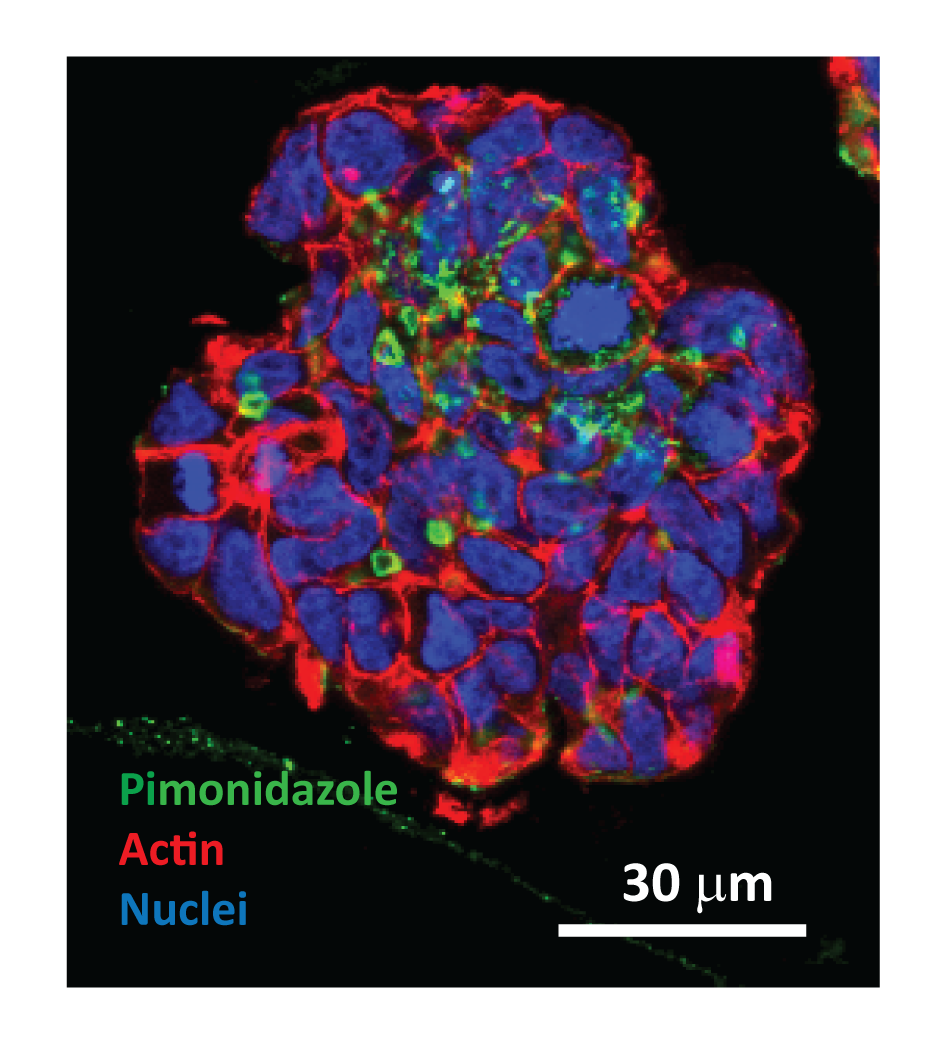

Supplement: Figure S4 — Immunofluorescent staining of LNCaP cells grown in the 2% PEG-based hydrogels for 14 days. Cultures were pretreated with 100 µm Pimonidazole hydrochloride for 4 h before being harvested and fixed for cryosectioning. Pimonidazole (green) is detected in a small region of the LNCaP colony indicating presence of hypoxic cells. CLSM images were taken at 40× magnification and 1.25 NA. Scale bar: 30 µm. (TIF) [file pone.0040217.s004.tif]

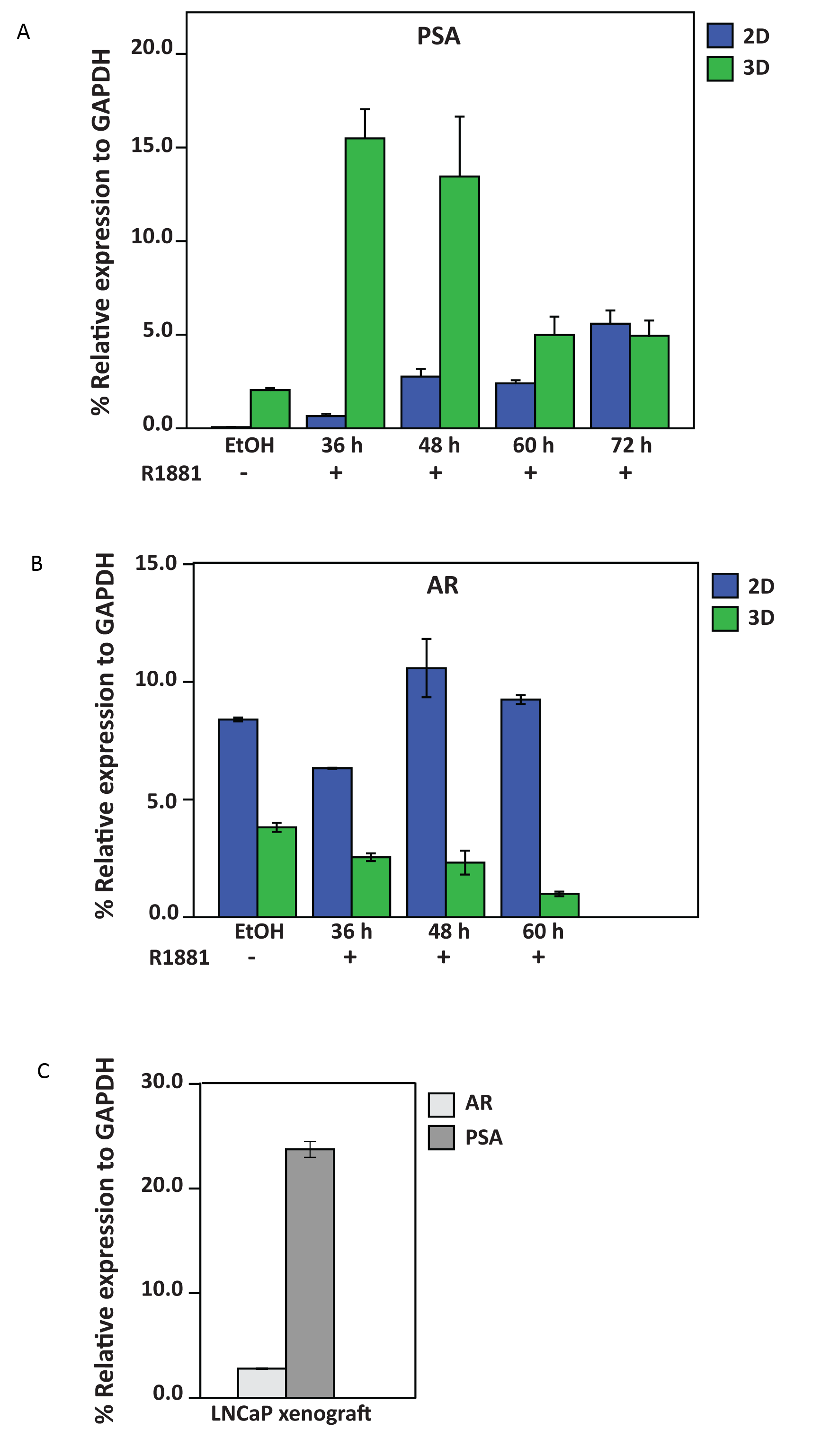

Supplement: Figure S5 — qRT-PCR of LNCaP cells treated with 1 nM R1881 in 2D and 3D cultures, and LNCaP xenograft. (A) mRNA level of PSA in 2D and 3D cultures at different treatment durations shows maximum induction for 3D cultures at 36–48 h and 48–72 h for 2D cultures. (B) mRNA level of AR changes with the treatment duration in both cultures. The expression decreased at 36 h of R1881 treatment in 2D cultures compared to non-treated (ethanol) controls but increased thereafter. In 3D cultures, a slight suppression was observed as the treatment was prolonged for more than 48 h. (C) The LNCaP xenograft from (after 12 weeks of inoculation) intact NOD/SCID mice shows comparable AR and PSA expression levels to 3D cultures treated with 1 nM R1881 for 48 h. (TIF) [file pone.0040217.s005.tif]

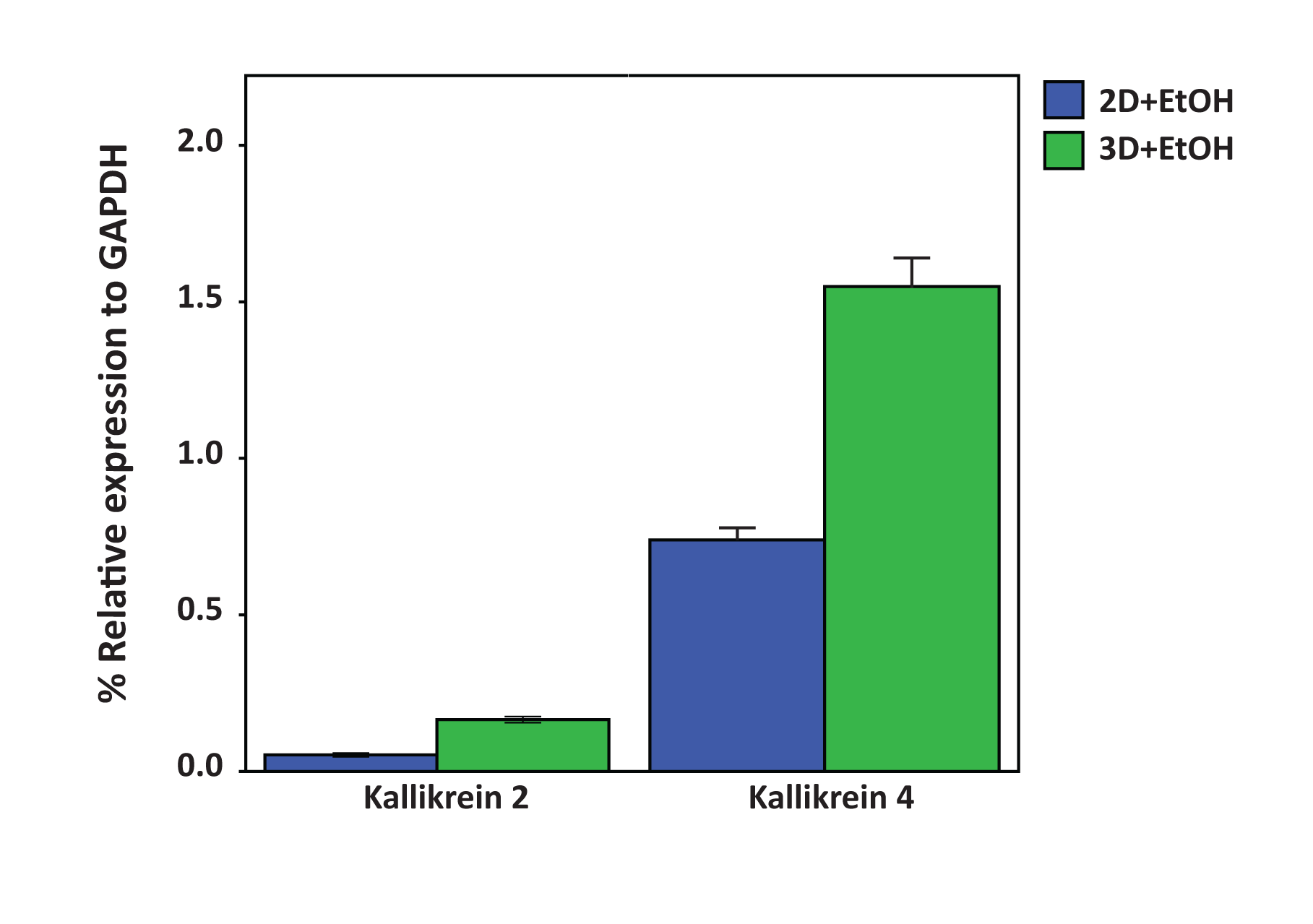

Supplement: Figure S6 — qRT-PCR of LNCaP cells under androgen deprived condition in 2D and 3D cultures. LNCaP cells were cultured in RPMI+5% CSS for 48 h followed by another 48 h in RPMI+5% CSS+ EtOH (0.08%) before being harvested for RNA isolation. Expression of Kallikrein 2 and Kallikrein 4 are both significantly higher in 3D compared to 2D cultures. (TIF) [file pone.0040217.s006.tif]
